# Ascl1b and Neurod1, instead of Neurog3, control pancreatic endocrine cell fate in zebrafish

**DOI:** 10.1186/1741-7007-11-78

**Published:** 2013-07-08

**Authors:** Lydie C Flasse, Justine L Pirson, David G Stern, Virginie Von Berg, Isabelle Manfroid, Bernard Peers, Marianne L Voz

**Affiliations:** 1 Laboratory of zebrafish development and disease models, University of Liege (ULg), 1, Giga-R, Avenue de l’Hopital, 1, Liege 4000, Belgium

**Keywords:** Pancreas, Endocrine, Zebrafish, Ascl1b, Neurod1, Neurog3

## Abstract

**Background:**

NEUROG3 is a key regulator of pancreatic endocrine cell differentiation in mouse, essential for the generation of all mature hormone producing cells. It is repressed by Notch signaling that prevents pancreatic cell differentiation by maintaining precursors in an undifferentiated state.

**Results:**

We show that, in zebrafish, *neurog3* is not expressed in the pancreas and null *neurog3* mutant embryos do not display any apparent endocrine defects. The control of endocrine cell fate is instead fulfilled by two basic helix-loop-helix factors, Ascl1b and Neurod1, that are both repressed by Notch signaling. *ascl1b* is transiently expressed in the mid-trunk endoderm just after gastrulation and is required for the generation of the first pancreatic endocrine precursor cells. Neurod1 is expressed afterwards in the pancreatic anlagen and pursues the endocrine cell differentiation program initiated by Ascl1b. Their complementary role in endocrine differentiation of the dorsal bud is demonstrated by the loss of all hormone-secreting cells following their simultaneous inactivation. This defect is due to a blockage of the initiation of endocrine cell differentiation.

**Conclusions:**

This study demonstrates that NEUROG3 is not the unique pancreatic endocrine cell fate determinant in vertebrates. A general survey of endocrine cell fate determinants in the whole digestive system among vertebrates indicates that they all belong to the ARP/ASCL family but not necessarily to the Neurog3 subfamily. The identity of the ARP/ASCL factor involved depends not only on the organ but also on the species. One could, therefore, consider differentiating stem cells into insulin-producing cells without the involvement of NEUROG3 but via another ARP/ASCL factor.

## Background

The pancreas is a mixed gland of the digestive tract composed of an exocrine compartment (acini and ducts), releasing digestive enzymes into the duodenum, and an endocrine compartment, secreting hormones into the bloodstream in order to control glucose homeostasis. Loss or dysfunction of endocrine insulin-secreting β-cells leads to diabetes, a widespread disease affecting more than 370 million people worldwide.

Outstanding progress has been made to set up new therapies for diabetes through cell therapy (reviewed by [1-5]). Recent efforts have been focused on directing stem cells to differentiate *in vitro* into pancreatic β cells that could be transplanted to diabetic patients [6]. To achieve that goal, it is essential to understand in detail the molecular mechanisms controlling pancreatic endocrine cell differentiation.

Although much of our knowledge on pancreas organogenesis relies on mouse genetic studies, the use of zebrafish has also significantly contributed to the deciphering of mechanisms involved in the earliest phases of pancreas development [7-12]. In this fish, the endoderm forms two converging sheets of cells by the end of gastrulation (10 hours post fertilization, hpf). Subsequently, these cells condense at the midline to form the endodermal rod which will give rise to the digestive tract and the associated organs [9,13]. Early in development, at the 10 somite stage (10s, 14 hpf), the homeobox Pdx1 factor starts to be expressed in the endodermal region located between the first and the fourth somite [14]. As in mammals, a dorsal and a ventral pancreatic bud will emerge from this *pdx1*+ region and will later coalesce to form the pancreas [15]. In zebrafish, the first hormone-expressing cells that appear from the dorsal bud are the insulin-producing β-cells, detected from 15 hpf onward. Next appear the somatostatin-secreting δ-cells (17 hpf), the ghrelin ϵ-cells (18 hpf) and finally the glucagon-producing α-cells (21 hpf). This first wave of endocrine cells is followed by a second wave coming from the ventral bud that forms from 32 hpf onwards [15-17]. After that stage, the increase of the endocrine cell mass is believed to result from the differentiation and proliferation of late forming ventral bud-derived endocrine cells [16]. Therefore, while at 2 days post fertilization (dpf), the vast majority of the endocrine cells is generated from the dorsal bud, at 12 days, a majority seems to derive from the ventral bud [16].

Both in zebrafish and mice, the Notch signaling pathway tightly controls pancreatic cell differentiation. Notch prevents commitment to the endocrine cell fate, thereby reserving a population of undifferentiated precursor cells for ongoing proliferation and generation of later-appearing cell lineages [18-21]. Notch pathway is a fundamental and evolutionarily conserved process in metazoan development. There are numerous core players that participate in this process. Briefly, via the Hairy Enhancers-of-split proteins, Notch signaling represses the expression of genes of the *Achaete-Scute* like (ASCL) family or of the *Atonal* related protein (ARP) family, this latter being subdivided into Atonal, Neurogenin and Neurod subfamilies. These genes encode basic helix-loop-helix (bHLH) transcription factors and can be classified in two categories, ‘cell fate determinant’ factors and ‘cell differentiation’ factors [22]. The cell fate determinants are transiently expressed at early stages and are both necessary and sufficient to initiate the development of a specific cell lineage. The ‘cell differentiation’ factors are expressed at later stages and implement the differentiation program initiated by the cell fate determinants. For example, in the murine pancreas, NEUROG3 is the cell fate determinant of the pancreatic endocrine lineage [23] as its transient expression initiates the endocrine differentiation program of all endocrine cells [24-26]. Indeed, almost no endocrine pancreatic cells were detected in the Neurog3 knock-out mice [25]. NEUROG3 triggers the sustained expression of the ‘cell differentiation gene’ *Neurod1* that maintains the endocrine cell differentiation program [27,28]. Homozygous Neurod1 null mice notably have a striking reduction in the number of insulin-producing β cells and fail to develop mature islets [29].

The *neurog3* gene is found in the zebrafish genome but, surprisingly zebrafish *neurog3* mRNAs were not detected in the developing pancreas while they were detected in the hypothalamus and intestine [20,30]. In this study, we extensively analyzed *neurog3* expression during pancreas development and could not detect any expression at any stages in this tissue. The lack of Neurog3 function in the zebrafish pancreas was further confirmed by analyzing the phenotype of the recently identified sa211 *neurog3* null mutant. As neuronal or endocrine cell-fate commitment controlled by Notch is classically carried out via ARP/ASCL factors [31-33], we next searched for other ARP/ASCL factors acting downstream of Notch signaling that would promote the formation of pancreatic endocrine cells. Among the 14 ARP/ASCL factors identified in the zebrafish genome, only *ascl1b* and *neurod1* were found to be strongly expressed at early stages of endocrine cell differentiation. Knock-down analysis reveals that these factors have complementary roles in endocrine cell differentiation and that their simultaneous inactivation leads to a loss of all hormone-secreting cells. These two bHLH factors are, therefore, playing together a role analogous to that described for murine NEUROG3.

## Results

### *neurog3* is not expressed in the pancreatic dorsal bud and null *neurog3* mutant embryos do not display any apparent endocrine defects

**Figure 1:**
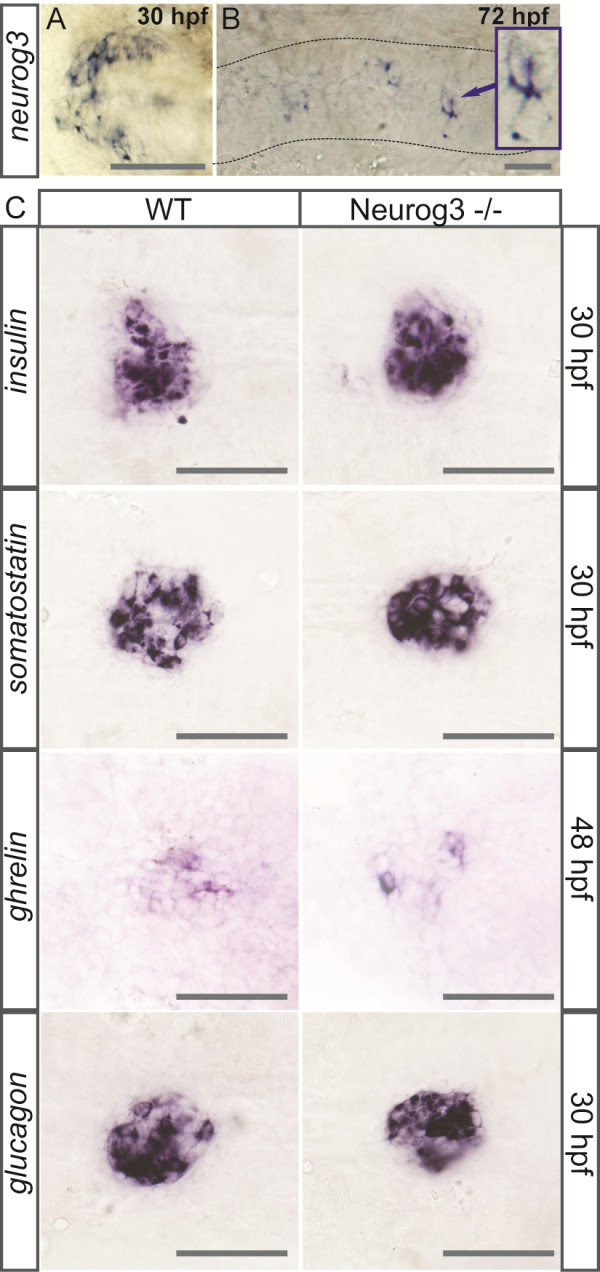
***neurog3 *is not expressed in the pancreas and null *neurog3 *mutant embryos do not display any apparent endocrine defects. (A-B)** Whole-mount *in situ* hybridization (WISH) showing expression of *neurog3* in the hypothalamus at 30 hpf **(A)** and in scattered cells of the gut (dotted lines) at 72 hpf **(B)**. **(C)** WISH showing that the number of cells expressing *insulin*, *somatostatin*, *ghrelin* and *glucagon* is not changed in the *neurog3* sa211 mutants compared to the *wt* siblings. All views are ventral with the anterior part to the left. Scale bars : 50 µm hpf, hours post fertilization; wt, wild type.

To determine whether the fundamental role of Neurog3 in endocrine cell differentiation in mice is conserved in zebrafish, we first analyzed *neurog3* expression in the pancreas by whole-mount *in situ* hybridization (WISH). *neurog3* expression was never detected in the pancreas at any developmental stages tested (14s, 18s, 24 hpf, 30 hpf, 52 hpf, 72 hpf, 4 dpf and 5 dpf) whereas its expression was found in the hypothalamus and in scattered cells of the intestine (Figure 1A-B), as reported previously [20,30]. To ensure that Neurog3 is actually not involved in pancreatic endocrine cell differentiation as *neurog3* expression level could be below the WISH detection limit, we analyzed the pancreas of a novel zebrafish *neurog3* mutant recently identified at the Zebrafish Mutant Resource [34]. This *neurog3* mutant allele (sa211) harbors an A to T substitution changing codon 74 (Arg) to a premature stop codon and is predicted to give rise to a truncated Neurog3 protein lacking the whole bHLH domain. The sa211 should be, therefore, a null allele. *neurog3* homozygous mutant embryos do not display any modifications in the number of pancreatic endocrine α-, β-, δ- and ϵ-cells compared to wild type embryos (Figure 1C). The same result was obtained when we knocked-down *neurog3* expression with a morpholino targeting its translation start site (Mo1) or its 5′UTR (Mo2) [see Additional file 1: Figure S1]. As extensive searches in the zebrafish genome did not identify any other *neurog3* paralog (see below), these data indicate that the zebrafish Neurog3 factor does not control pancreatic endocrine cell fate, in contrast to the murine NEUROG3, and suggest that another bHLH factor is playing its role in this fish model.

### Expression of ARP/ASCL genes in the developing zebrafish pancreas

**Figure 2:**
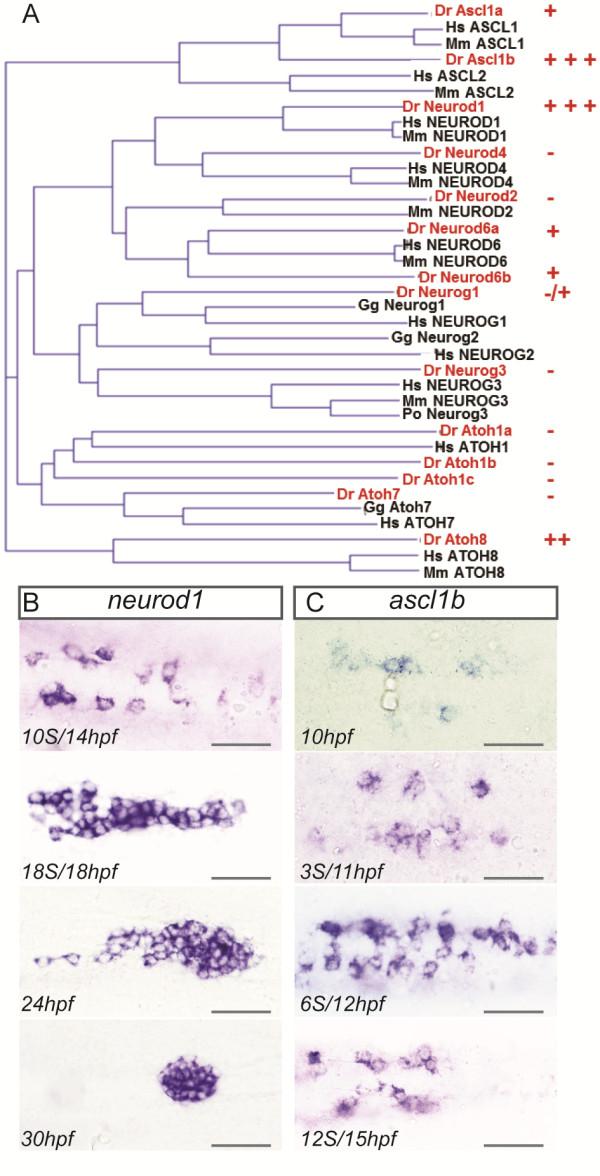
***ascl1b *and *neurod1 *are expressed during early pancreas development. (A)** Phylogenetic tree calculated with full-length amino acid sequences from vertebrate members of the ARP/ASCL family. The pancreatic expression of zebrafish ARP/ASCL was tested by WISH and the expression levels are depicted by (+++) highly, (++) moderately, (+) weakly, (-/+) very weakly and (-) not expressed in the pancreas. **(B-C)** WISH showing expression of *neurod1***(B)** and *ascl1b***(C)** in the pancreatic region at the indicated stages. All views are ventral with anterior part to the left. Scale bars : 50 µm WISH, whole-mount *in situ* hybridization.

In order to identify the functional equivalent of murine NEUROG3 in zebrafish, we searched for another ARP/ASCL gene that could assume its role. For that purpose, we performed a phylogenetic analysis of the various ARP/ASCL found in the zebrafish genome based on the bHLH directory described by Wang *et al*. [35] (Figure 2A). This allowed us to identify 14 ARP/ASCL genes in the zebrafish genome, including *neurog3* and another *neurogenin* gene, *neurog1*. Extensive *in silico* searches did not identify any other neurogenin genes in the zebrafish genome (see Material and Methods)*.*

The expression of the 14 ARP/ASCL genes was analyzed by WISH at different time points during pancreas development (6 to 8s, 12 to 14s, 18 to 20s, 24 hpf, 30 hpf, 48 hpf, 72 hpf). We observed high expression in the pancreatic region at early stages for only two factors, namely *ascl1b* and *neurod1* (Figure 2B-C). In contrast, only weak expression was detected at early stages for *ascl1a, neurog1* and *neurod6b* within the pancreatic area [see Additional file 2: Figure S2, A-C], while *atoh8* and *neurod6a* genes were detected only at late stages, that is, around two dpf [see Additional file 2: Figure S2, D-E].

*neurod1* expression has already been reported in the dorsal pancreatic bud in zebrafish [20,36-38] but its precise expression profile was not determined. In this study, we show that pancreatic *neurod1* expression starts around 6s (12 hpf) in a few cells. At 10s (14 hpf), *neurod1* is expressed in two rows of cells located on both sites of the midline (Figure 2B). Over the next four hours, the number of *neurod1+* cells progressively increases and they start to cluster to form the islet which is completely formed at 30 hpf. *neurod1* remains expressed in the pancreas of four dpf larvae as well as in adults (data not shown).

*ascl1b* is detected before *neurod1* as its expression starts as early as 10 hpf (bud stage) in two rows of cells in the prospective pancreatic region (Figure 2C). Over the following two hours, the number of *ascl1b*-expressing cells increases to reach its maximal level around 12 hpf. Then, *ascl1b* expression progressively decreases and is turned off at 17 hpf.

### *ascl1b* and *neurod1* are both expressed in the pancreatic endocrine precursors and their expression is controlled by Notch signaling

**Figure 3:**
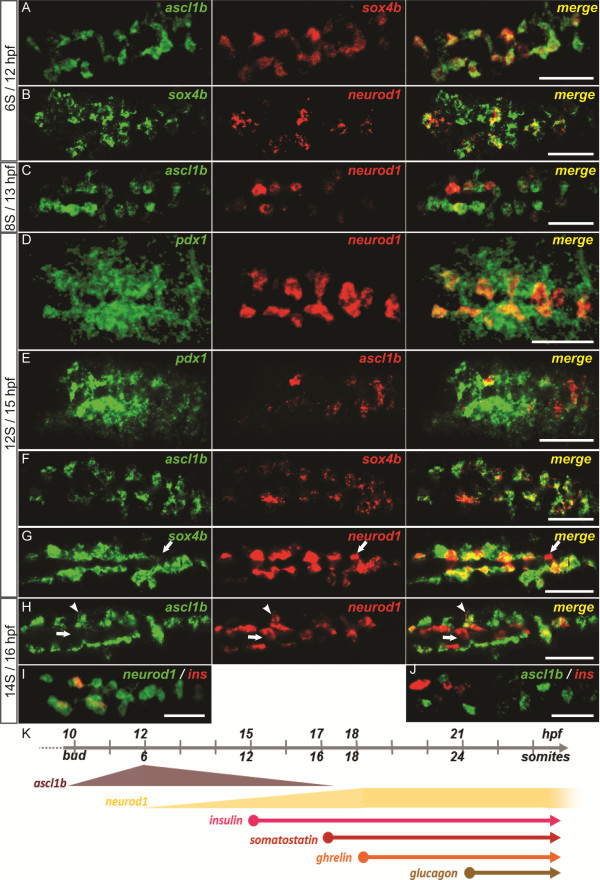
***ascl1b *and *neurod1 *are both expressed in pancreatic endocrine precursors. (A-J)** Double fluorescent WISH comparing the expression of *ascl1b* and *neurod1* with the expression of the pancreatic factor *sox4b***(A, B, F, G)**, *pdx1***(D, E)** or with the insulin hormone **(I, J)** at early stages (12 to 16 hpf). The arrows point to a cell *neurod1*+/*sox4b*- **(G)** or *neurod1*+/*ascl1b*- **(H)**. The arrowheads point to a cell *neurod1+/ascl1b+***(H)**. Z-plane confocal images. All views are ventral with anterior part to the left. **(K)** Diagram illustrating the time windows for the expression of *ascl1b*, *neurod1* and the pancreatic hormones. Scale bars : 50 µm hpf, hours post fertilization; WISH, whole-mount *in situ* hybridization.

In order to determine in which pancreatic cell types these two bHLH factors are expressed, double fluorescent WISH was performed using probes for other pancreatic factors. As mentioned above, *ascl1b* expression starts at the end of gastrulation (10 hpf, bud stage) in the prospective pancreatic region where *sox4b*, the first known marker of endocrine precursors, will appear about two hours later [37,38]. As soon as *sox4b* is expressed in the pancreatic anlagen, its expression domain overlaps almost perfectly with *ascl1b* (Figure 3A) and these two factors remain co-expressed over the next five hours (Figure 3F and data not shown) until the expression of *ascl1b* switches off at 17 hpf. At 12 hpf, *neurod1* starts to be expressed within the *sox4b/ascl1b* expression domain (Figure 3B and C). At 14 hpf, *pdx1* expression is initiated in two bilateral rows of endodermic cells adjacent to the midline [14]. *ascl1b* and *neurod1* are expressed within the medial part of the *pdx1* expression domain, which includes the pancreatic endocrine precursors (Figure 3D and E) [8,9]. From 15 hpf, the two stripes of pancreatic precursors have begun to coalesce toward the midline starting from the posterior part (Figure 3F and G) [14,37]. At this stage, *neurod1*+ cells are still largely included in the *ascl1b/sox4b* domain with the exception of a few cells which are only labeled by *neurod1* (arrow in Figure 3G). One hour later, the segregation of *neurod1+* cells from the *ascl1b/sox4b* endocrine precursor cells is more striking (Figure 3H). This separation coincides with the appearance of the first hormone-expressing cells (that is, insulin-producing cells*)* which co-express *neurod1* but not *ascl1b* (Figure 3I, J). At 17 hpf, the expression of *ascl1b* turns off. In contrast, *neurod1* expression persists and, as shown previously [38], at 24 hpf *neurod1* labels the whole endocrine cell lineage including the *sox4b*+ endocrine precursor cells and the hormone-expressing cells. At 30 hpf, when the majority of endocrine cells are differentiated and clustered into the single islet, *neurod1* remains expressed in all endocrine cell types (α, β, δ and ϵ) (data not shown). All these data show that *ascl1b* is transiently expressed in the pancreatic endocrine precursors and is the earliest pancreatic marker identified so far (Figure 3K). *neurod1* expression is initiated two hours later in the endocrine precursors and remains expressed in the mature endocrine cells, in contrast to *ascl1b*.

**Figure 4:**
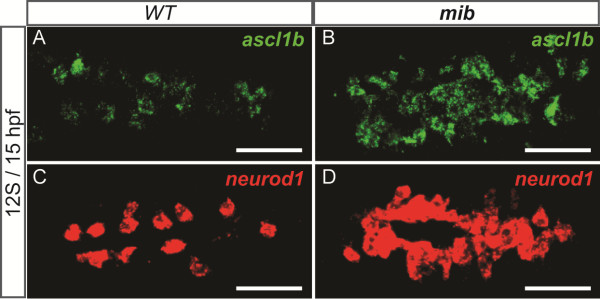
**Pancreatic expression of *ascl1b *and *neurod1 *is repressed by Notch signaling.** Fluorescent WISH showing pancreatic expression of *ascl1b***(A-B)** and *neurod1***(C-D)** in *wild-type* (*wt)* embryos **(A, C)** and *mind bomb* (*mib)* mutants **(B, D)**. Confocal projection images. All views are ventral views of 15 hpf embryos with anterior part to the left. Scale bars : 50 µm hpf, hours post fertilization; WISH, whole-mount *in situ* hybridization.

We next asked whether *ascl1b* and *neurod1* could be mediators of Notch signaling by analyzing their expression in *mind bomb (mib)* mutants. *mib* mutants lack a Delta-ubiquitin ligase, resulting in the failure to trigger Delta-mediated Notch signaling [39] and leading to an increase of endocrine differentiation at early stages [20,37]. We found that *neurod1* and *ascl1b* expression is strongly increased in *mib* pancreas (Figure 4), indicating that *ascl1b* and *neurod1* expression is repressed by Notch signaling, further suggesting a role of these two factors in endocrine differentiation.

### Impaired endocrine cell differentiation in *neurod1* morphants

**Figure 5:**
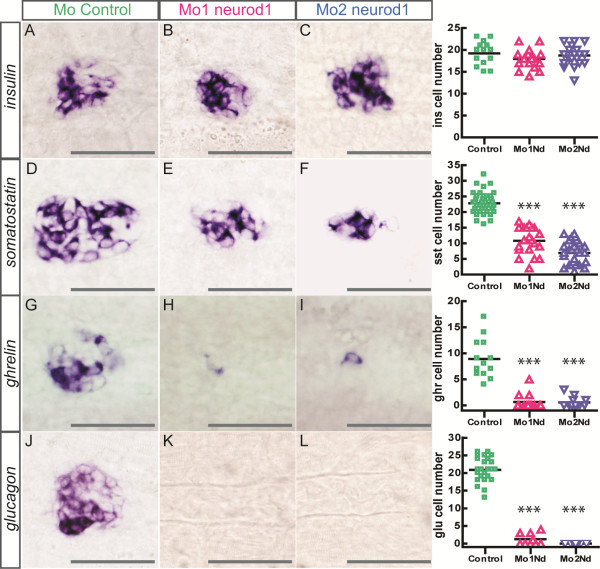
**Impaired endocrine cell differentiation in *neurod1 *morphants.** Ventral views with anterior to the left of WISH showing expression of *insulin***(A-C)**, *somatostatin***(D-F)**, *ghrelin***(G-I)** and *glucagon***(J-L)** in control **(A, D, G, J)**, Mo1-*neurod1***(B, E, H, K)** and Mo2-*neurod1***(C, F, I, L)** morphants at 30 hpf. The quantifications on the right side of the figure represent the number of positive cells per embryo for controls and *neurod1* morphants. Asterisks (***) indicate that the difference between cell number in controls and *neurod1* morphants is statistically significant by Student’s t-test (*P* <0.001). Scale bars : 50 µm hpf, hours post fertilization; WISH, whole-mount *in situ* hybridization.

To assess the role of Neurod1 in pancreatic endocrine development, we abrogated Neurod1 protein expression in zebrafish embryos by injecting two distinct antisense morpholinos. The first (Mo1-*neurod1*) targets the translational start site while the second (Mo2-*neurod1*) targets the 5′UTR region. Both morpholinos efficiently block the translation of the *neurod1* transcript as they completely prevent the expression of GFP from the Tg(*neurod1:egfp*) transgenic line [40], while they do not disturb the overall morphology of the embryos [see Additional file 3: Figure S3]. In contrast, Neurod1 knockdown leads to pancreatic defects as the injection of each morpholino led to the same phenotype: an almost complete depletion of *glucagon* (α) and *ghrelin* (ϵ) cells (Figure 5G-L) together with a severe reduction in the number of *somatostatin* cells (δ) (Figure 5D-F). In contrast, the number of *insulin* cells (β) was not significantly decreased (Figure 5A-C). As the Mo2 morpholino was slightly more efficient than Mo1, the following experiments were mainly performed with Mo2.

**Figure 6:**
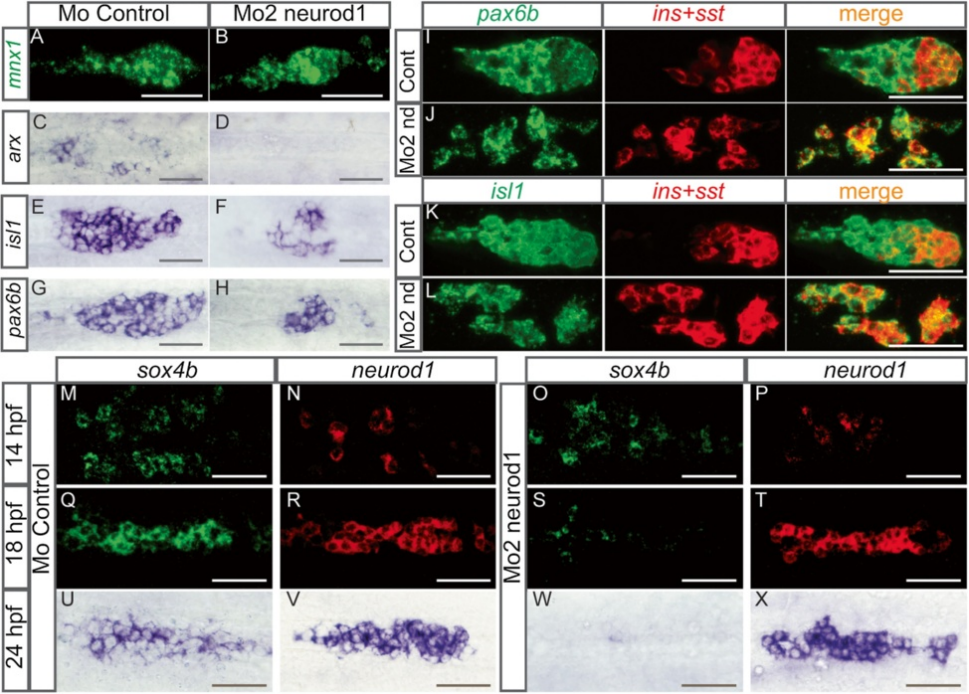
**Pancreatic cells are arrested in their differentiation process in *neurod1 *morphants and the expression of *sox4b *is not maintained.** Ventral views with the anterior to the left of the pancreas of embryos analyzed by visible **(C-H, U-X)** or fluorescent **(A-B, I-T)** WISH. **(A-H)** Pancreatic expression of *mnx1*, *arx*, *isl1* and *pax6b* in controls and Mo2-*neurod1* morphants at 24 hpf, the number of *isl1*+ cells in control morphant (50.4 ± 2.1) was reduced about 2.1 fold in the Mo2-*neurod1* morphant (24.1 ± 1.2) and the number of *pax6b*+ cells in control morphant (40.7 ± 1.2) was reduced about 1.8 fold in the Mo2-*neurod1* morphant (22.1 ± 1.2). **(I-L)** Double fluorescent WISH performed with *pax6b***(I, J)** or *isl1***(K,L)** probe together with a mix of *insulin* (*ins)* and *somatostatin* (*sst)* probes on 24 hpf controls or Mo2-*neurod1* morphants. Z-plane confocal images **(M-T)** Double fluorescent WISH performed with *sox4b* and *neurod1* on 14 hpf and 18 hpf controls or Mo2-*neurod1* morphants. Confocal projection images **(U-X)** WISH performed with *sox4b* and *neurod1* on 24 hpf controls or Mo2-*neurod1* morphants. Scale bars : 50 µm hpf, hours post fertilization; WISH, whole-mount *in situ* hybridization.

To understand the mechanism by which Neurod1 controls endocrine cell differentiation, we analyzed the expression of various pancreatic transcription factors in the *neurod1* morphants at 24 hpf. Consistent with the loss of α-cells, we observed a complete extinction of *arx,* a factor known to be expressed specifically in all α-cells and essential for their differentiation [41] (Figure 6C and D). In contrast, expression of the *mnx1/hb9* gene, specifically involved in β-cell differentiation [36,42] was unchanged, like *insulin* (Figure 6A and B). The expression domain of *pax6b* and *isl1*, which at 24 hpf includes all differentiated hormonal cells [38,43] was clearly reduced (around two-fold, Figure 6E-H), consistent with α-, ϵ-cells loss and a reduced number of δ-cells. Cells still expressing these two factors correspond to β-cells and to the residual δ-cells still present in *neurod1* morphants (Figure 6J-L). Since the morpholinos are designed to prevent *neurod1* mRNA translation but not the expression of the transcripts, we could highlight *neurod1*+ cells by WISH in the *neurod1* morphants. *neurod1*+ cells were present in *neurod1* morphants at all stages tested (14 hpf, 18 hpf, 24 hpf and 30 hpf) and we did not detect any obvious changes in their number compared to the control morphants (Figure 6P, T, X and data not shown). This suggests that the loss of α cells in the *neurod1* morphants is not due to the apoptosis of the *neurod1*+ cells but due to a blockage in their differentiation process. This was further confirmed by performing terminal deoxynucleotidyl transferase dUTP nick end labeling (TUNEL) assays revealing no apoptotic cells in the pancreatic area of *neurod1* and control morphants at different stages (26, 30, 40, 48 and 55 hpf) [see Additional file 4: Figure S4 A-D and data not shown].

To further define *neurod1* function in early steps of endocrine cell differentiation, we analyzed the expression of *sox4b* at early stages of development. While the initiation of *sox4b* expression is not perturbed in *neurod1* morphants (Figure 6M and O) its expression is strongly reduced at 18 hpf (Figure 6Q and S) and is not detectable anymore at 24 hpf (Figure 6U and W). All together, these data show that, in the absence of Neurod1, *sox4b* expression is correctly initiated. Then, around 17 to 18 hpf, the stage when most β-cells and a minority of δ-cells are already differentiated (see diagram in Figure 3K), *sox4b* expression is no longer maintained and the differentiation of late-appearing endocrine cells is blocked, leading to a loss of ϵ- and α-cells as well as to a reduction of δ-cells.

### Impaired endocrine cell differentiation in *ascl1b* morphants

Intriguingly, although *neurod1* is expressed from 12 hpf (6s) in the pancreatic endocrine precursors, *neurod1* morphants do not display any apparent pancreatic defects before 17 hpf. We, therefore, hypothesized that, at early stages, Ascl1b could complement the loss of Neurod1 function. To test this hypothesis, we first defined Ascl1b function in endocrine differentiation by injecting an antisense morpholino targeting the translational start site of *ascl1b* mRNA as described in [44,45].

**Figure 7:**
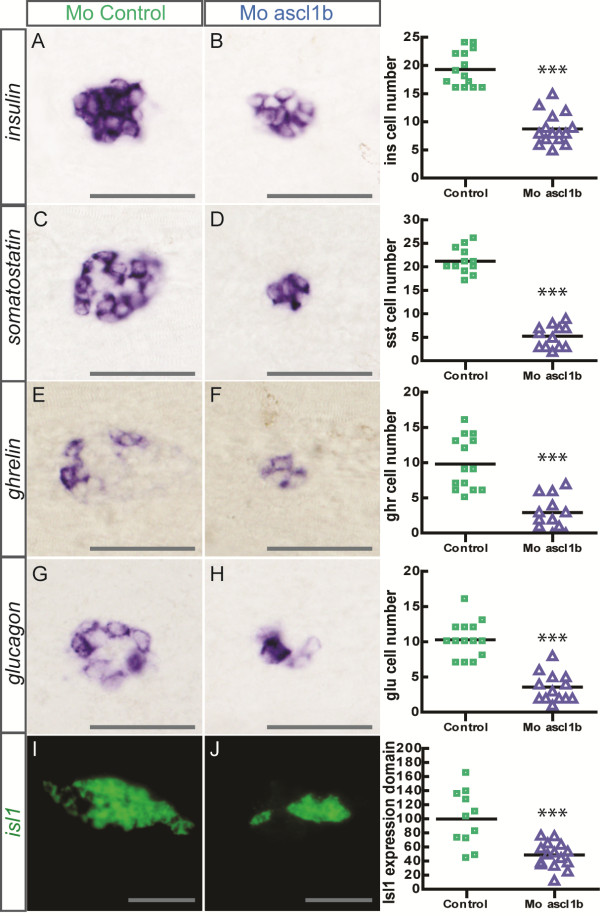
**Impaired endocrine cell differentiation in *ascl1b *morphants.** Ventral views with the anterior part to the left of WISH showing expression of *insulin***(A-B)**, *somatostatin***(C-D)**, *ghrelin***(E-F)** and *glucagon***(G-H)** in control **(A, C, E, G)** and *ascl1b* morphants **(B, D, F, H)** at 30 hpf. The quantifications on the right side of the figure represent the number of positive cells per embryo for controls and *ascl1b* morphants. **(I-J)** Confocal projections of ventral views of fluorescent WISH showing expression of *isl1* in control **(I)** and *ascl1b* morphants **(J)** at 24 hpf. The quantification of *isl1* expression domain is depicted on the right side of the figure. The graph shows the relative volume of *isl1+* cells in control and *ascl1b* morphants, the mean of the volume occupied by the *isl1*+ cells in control morphants being arbitrarily set to 100%. Asterisks (***) indicate that the difference between controls and *ascl1b* morphants is statistically significant by Student’s t-test (*P* <0.001). Scale bars : 50 µm hpf, hours post fertilization; WISH, whole-mount *in situ* hybridization.

In contrast to *neurod1* morphants, in *ascl1b* morphants we observed a significant reduction in the number of all types of pancreatic hormone-expressing cells (Figure 7). Consistent with this phenotype, we noticed a reduction of the *isl1* expression domain, which includes all the mature endocrine cells at 24 hpf (Figure 7I, J). As *ascl1a*, the paralog of *ascl1b*, was detected in a few cells of the endocrine pancreas at 18 hpf [see Additional file 2: Figure S2B], we tested if Ascl1a could have redundant functions with Ascl1b. This is not the case as the knockdown of Ascl1b in the *ascl1a*/*pia* null mutant [46] did not lead to a further reduction of endocrine cells (data not shown).

**Figure 8:**
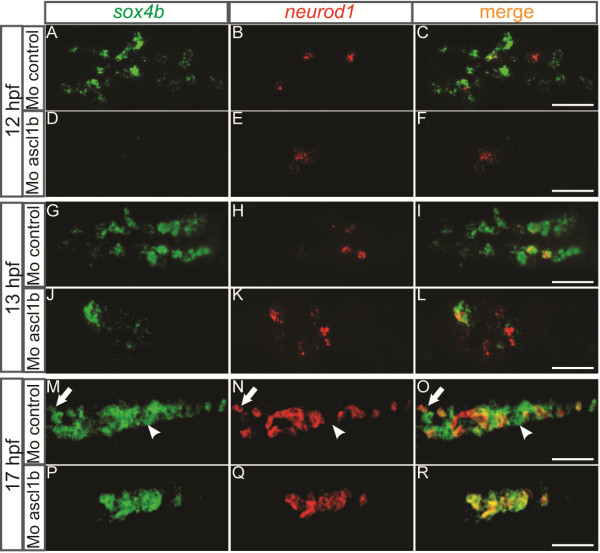
**The onset of *sox4b *expression is delayed in *ascl1b *morphants and is restricted to *neurod1 *expressing cells.** Ventral views with the anterior part to the left of double fluorescent WISH showing expression of *sox4b* and *neurod1* at 12 hpf **(A-F)**, 13 hpf **(G-L)** and 17 hpf **(M-R)** in control and *ascl1b* morphants. The domain occupied by *sox4b*+ and *neurod1+* cells was reduced respectively 3.3- and 2.6-fold in the Mo-*ascl1b* at 17 hpf. The arrows point to a cell expressing *sox4b* and *neurod1* while the arrowheads points to a cell expressing only *sox4b*. Z-plane confocal images. Scale bars : 50 µm hpf, hours post fertilization; WISH, whole-mount *in situ* hybridization.

To further define Ascl1b function, we analyzed the initiation of endocrine cell differentiation in *ascl1b* morphants. At 12 hpf (6s), we could not detect any *sox4b*+ cells in the *ascl1b* morphants whereas *sox4b* was easily detectable in control morphants (Figure 8A and D). At 13 hpf (8s), one or two *sox4b*+ cells were present in the *ascl1b* morphants (Figure 8J), and over the next four hours, new *sox4b*+ cells appeared but their number never reached the control levels (30% compared to the control at 17 hpf, Figure 8P). In contrast, *neurod1* expression was correctly initiated in *ascl1b* morphants (Figure 8E and K). Interestingly, the few *sox4b+* cells detected in *ascl1b* morphants were all seen within the *neurod1*+ domain (Figure 8L and R) while in control morphants, a majority of *sox4b+* cells do not express *neurod1* (Figure 8C, I and O). These data suggest that, in *ascl1b* morphants, *sox4b* gene expression is switched on by Neurod1. This hypothesis is in accordance with the onset of *sox4b* that is detected just after *neurod1* activation in *ascl1b* morphants.

### Endocrine precursor differentiation is abolished in the double *ascl1b* and *neurod1* morphants

**Figure 9:**
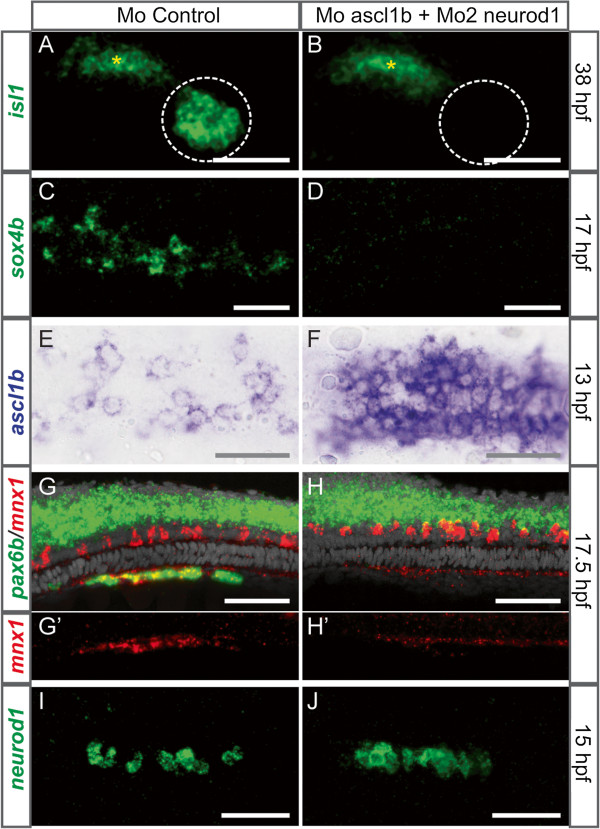
**Endocrine cell differentiation is abolished in the double *ascl1b *and *neurod1 *morphants. **Ventral views of WISH showing expression of *insulin***(A-B, I-J)**, *somatostatin***(C-D)**, *ghrelin***(E-F)** and *glucagon***(G-H)** in control **(A,C,E,G,I)** and Mo-*ascl1b*/Mo2-*neurod1***(B,D,F,H,J)** morphants at 38 hpf **(A-H)** or 17 hpf **(I-J)**. The quantifications on the right side of the figure represent the number of positive cells per embryo for controls and double *ascl1b/neurod1* morphants. Asterisks (***) indicate that the difference between cell number in controls and double *ascl1b/neurod1* morphants is statistically significant by Student’s t-test (*P* <0.001). hpf, hours post fertilization; WISH, whole-mount *in situ* hybridization.

**Figure 10:**
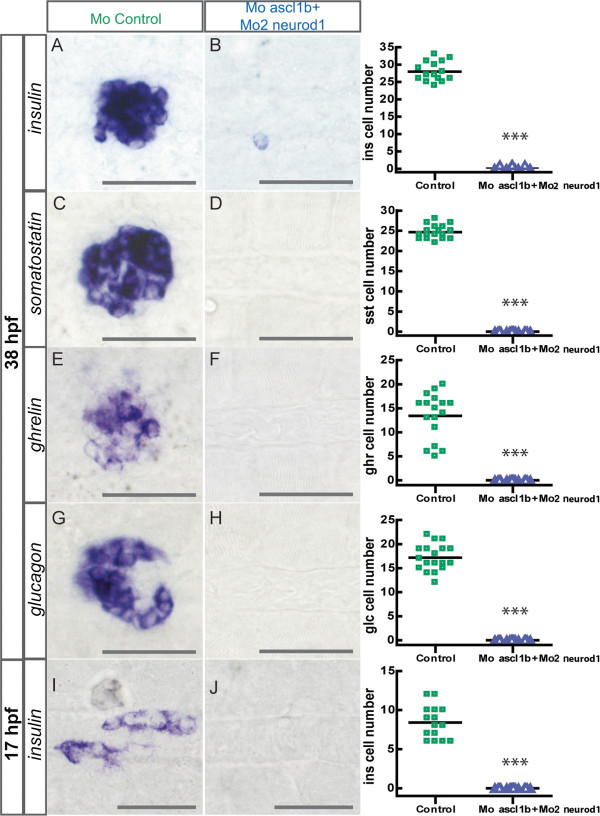
**Simultaneous knock-down of Ascl1b and Neurod1 blocks the differentiation process of the precursors.** Ventral views with the anterior part to the left of the pancreas of control **(A,C,E,G,I)** or Mo-*ascl1b*/Mo2-*neurod1* morphants **(B,D,F,H,J)** analyzed by fluorescent **(A-D, G-J)** or visible **(E-F)** WISH. **(A-B)** Confocal projections of *isl1* expression in the pancreatic islet (dotted line) and in the adjacent mesoderm (*) at 38 hpf **(C-D)** and of *sox4b* expression at 17 hpf. **(E-F)***ascl1b* transcript expression at 13 hpf (8s). **(G-H)** Confocal projections of *pax6b* (green) and *mnx1* (red) expression at 17.5 hpf showing that the pancreatic expression of *pax6b* and *mnx1* is completely lost in the double morphants. In contrast, the expression of *pax6b* in the neural tube and the expression of *mnx1* gene in the motoneurons and the hypochord is not perturbed. **(I-J)***neurod1* transcript expression at 15 hpf (12s). hpf, hours post fertilization; WISH, whole-mount *in situ* hybridization.

To test the hypothesis that *sox4b* expression can be switched on either through Ascl1b or Neurod1 action, we performed a co-injection of the morpholinos targeting both genes. As shown on Additional file 5: Figure S5, this co-injection does not affect the general morphology of the embryos. In these double *ascl1b/neurod1* morphants, *sox4b* expression was never detected, not at 17 hpf (Figure 9C-D) nor later on (data not shown). Moreover, this double knock-down abolished all endocrine cell differentiation as we did not detect any hormone-producing cells at early or late stages (17 or 38 hpf) (Figure 10A-J). In addition, *isl1* expression was not detected in the endocrine islet at 38 hpf while *isl1* was correctly expressed in the adjacent mesodermal tissue [10] (Figure 9A-B). Similarly, the expression of *pax6b* and *mnx1* was completely lost in the endocrine pancreas while *pax6b* remains expressed in the neural tube and *mnx1* gene in the motoneurons and the hypochord (Figure 9G-H). In contrast to these transcription factors, the *ascl1b* transcripts were still detected in the double morphants and the number of *ascl1b* positive cells was drastically increased at 13 hpf (Figure 9E-F) suggesting that these cells are blocked in their differentiation process. *neurod1* transcript was also detected in the double morphants (Figure 9I-J), confirming the above observations that *neurod1* initiation can occur independently of Ascl1b. As expected, based on the increase of the number of cells expressing the *ascl1b* transcripts, we did not observe any apoptosis at 14s (16 hpf) and 19s (18.5 hpf) in these double morphants [see Additional file 4: Figure S4E-H].

Altogether, our data indicate that the simultaneous loss of *ascl1b* and *neurod1* expression abolishes the formation of all pancreatic hormone-expressing cells by interfering with the initiation of their differentiation process as shown by the loss of the pancreatic expression of *sox4b, isl1, pax6b, mnx1* and *arx* genes.

### *ascl1b* and *neurod1* are also expressed in the endocrine cells of the ventral bud but not *neurog3*

**Figure 11:**
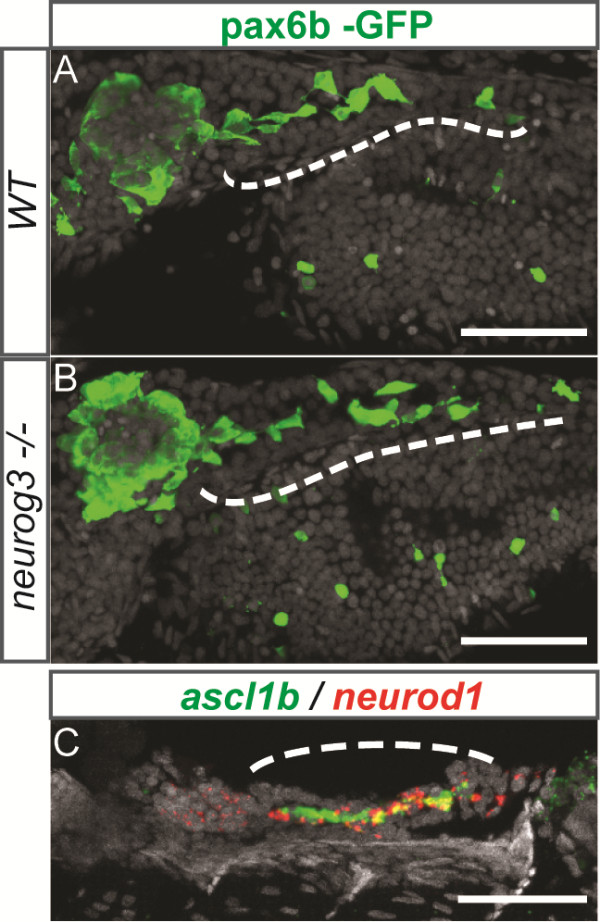
**Neurog3 is not involved in the formation of the second wave endocrine cells. (A-B)** Ventral views of immunohistochemistry against GFP of five dpf *wt***(A)** or *neurog3* null mutant **(B)** tg(pax6b:GFP) embryos treated from three to five dpf with the Notch signaling inhibitor LY411575. The generation of *pax6b* endocrine cells from the intrapancreatic duct (indicated by a dash line) is not perturbed in the *neurog3* null mutant (n = 6). **(C)** Confocal projections of *ascl1b* (green) and *neurod1* (red) expression of four dpf embryos treated from three to four dpf with the Notch signaling inhibitor LY411575 showing a strong expression in the intrapancreatic duct. Anterior part to the left. dpf, days post fertilization; wt, wild type.

As endocrine cells are also generated at later stages from the intrapancreatic ducts [16,17,47], we investigated whether Neurog3 could be involved in the generation of these late endocrine cells. To detect them more easily, we treated *wt* and *neurog3* null mutant embryos from three to five dpf with the Notch-signaling inhibitor LY411575, treatment known to drastically increase the number of endocrine cells coming from the intrapancreatic ducts (Figure 11A) [17,47,48]. As shown on Figure 11B, the homozygous *neurog3* mutants are able to generate endocrine cells efficiently from the intrapancreatic ducts (n = 6), suggesting that Neurog3 is not involved in the formation of the second wave endocrine cells, as it is in the first wave. This is in agreement with the absence of *neurog3* expression in the intrapancreatic ducts of LY411575-treated embryos (data not shown). In contrast, we detected a strong expression of *ascl1b* in the intrapancreatic ducts of the LY411575-treated embryos (Figure 11C) as well as of *neurod1* as previously described [48,49], suggesting that the couple Neurod1 and Ascl1b could also play an important role in the formation of second wave endocrine cells. The verification of this hypothesis will require the generation of *ascl1b* and *neurod1* null mutant as the knock-down of Neurod1 is no longer fully efficient after 48 hpf.

## Discussion

In this study, we show that, in zebrafish, Neurog3 does not control pancreatic endocrine cell fate as it does in the mouse. This function is fulfilled by two ASCL/ARP factors, Ascl1b and Neurod1.

**Figure 12:**
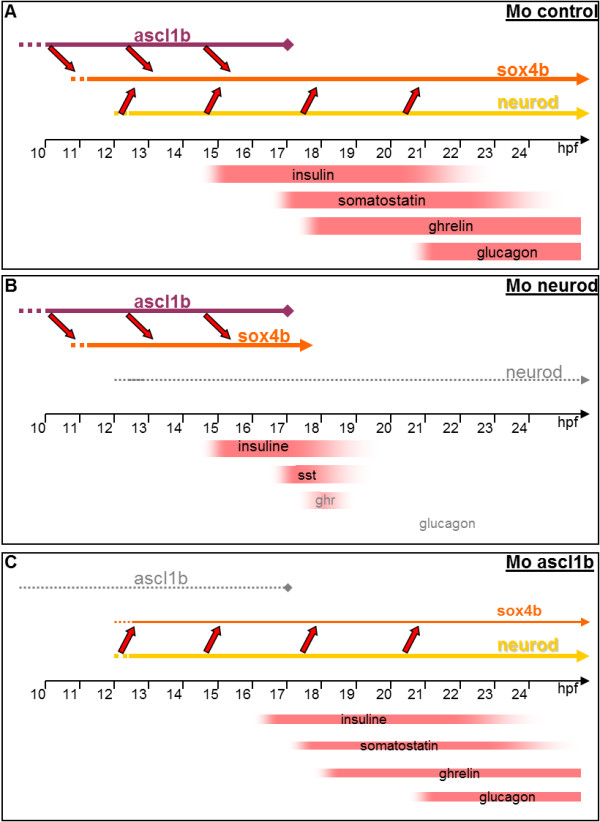
**Model describing the function of *ascl1b *and *neurod1 *in pancreatic endocrine differentiation. (A)** In *wt* embryos, Ascl1b initiates the expression of *sox4b* in the endocrine precursors at 11 hpf. Then, around 12 hpf, *neurod1* expression starts and maintains *sox4b* expression in the precursors. The differentiation of the distinct endocrine cell types can take place. **(B)** In *neurod1* morphants, Ascl1b initiates the expression of *sox4b* in the endocrine precursors at 11 hpf and maintains s*ox4b* expression until 17 hpf. From that stage, *ascl1b* expression turns off and *sox4b* expression is no longer maintained. The differentiation of the late-appearing endocrine cells is blocked. **(C)** In *ascl1b* morphants, *sox4b* expression is not initiated at 11 hpf. When Neurod1 starts to be expressed from 12 hpf, it initiates *sox4b* expression in a reduced number of endocrine precursors. hpf, hours post fertilization; wt, wild type.

Ascl1b starts to be expressed at bud stage (10 hpf) in the prospective pancreatic region about two hours before *sox4b*, that was previously the earliest known marker of endocrine precursors [37,38,50]. We show that Ascl1b initiates the pancreatic endocrine cascade and induces the formation of the first *sox4b*+ endocrine pancreatic precursors (see the model in Figure 12A). Two hours after *ascl1b* onset, *neurod1* expression appears within the *ascl1b/sox4b* domain. Neurod1 pursues the endocrine differentiation program initiated by Ascl1b notably by maintaining the expression of *sox4b* in the precursors and allowing their subsequent differentiation. Indeed, in Neurod1 morphants, *sox4b* expression is properly initiated by Ascl1b but, around 17 to 18 hpf, when *ascl1b* expression turns off, it is no longer maintained (Figure 12B). The endocrine differentiation does not proceed further, thereby preventing the differentiation of later endocrine cells, that is, the majority of δ (*somatostatin*), ϵ (*ghrelin*) and α (*glucagon*) cells, normally appearing, respectively, at 17 hpf, 18 hpf and 21 hpf (Figure 12B).

The role of Neurod1 as a ‘cell differentiation factor’ required for the maintenance of the endocrine differentiation program in zebrafish is similar to the murine situation where NEUROD1 pursues the endocrine differentiation program initiated by NEUROG3 and participates in the maintenance of the mature islet cells (reviewed by [51,52]). However in zebrafish, if Ascl1b is absent, Neurod1 remains expressed and acts as a cell fate determinant inducing the formation of the *sox4b+* precursors. Indeed, in *ascl1b* morphants, *sox4b+* precursor cells are detected only after *neurod1* onset and *sox4b* expression is exclusively induced in *neurod1* expressing cells while, in the control morphants, the majority of *sox4b+* cells do not express *neurod1*. Consequently, the pool of endocrine precursors is reduced in Ascl1b morphants, leading to a reduced number of all mature endocrine cells at 30 hpf (Figure 12C). Thus, in zebrafish, *neurod1* has the capacity to act as a ‘cell fate determinant’ or a ‘cell differentiation’ factor. In mice, NEUROD1 has been reported to act only as a cell differentiation factor. However, when NEUROD1 is ectopically expressed in the murine pancreatic anlagen under the control of the Pdx1 promoter, it has the same intrinsic capacity as Neurog3 to induce endocrine differentiation [28]. Such functional equivalence highlighted by a gain-of-function approach can probably be explained by the fact that these two factors regulate largely overlapping sets of genes [27].

This dual capacity to promote the selection of precursors and to regulate some differentiation steps is not restricted to Neurod1 but is shared by many ASCL/ARP factors. For example, in the olfactory epithelium, *Mash1* and *Neurog1* are expressed sequentially in the sensory neuron precursors. MASH1 acts as a cell fate determinant by inducing the olfactory precursors, while Neurog1 acts as a cell differentiation gene by allowing the differentiation of these precursors into sensory neurons. However, NEUROG1 can partly compensate for the loss of the determination function of MASH1 in the olfactory placode, suggesting that NEUROG1 also has the intrinsic capacities to act as a cell determinant or differentiation factor in the same tissue [53]. This suggests that the function of some ARP/ASCL factors is not intrinsically determined by their protein sequence and their structure but could be simply dictated by the context (cell type and timing of expression). This flexibility could be explained by the strong conservation within the bHLH domain. Nine of the ten residues predicted to contact DNA are identical among ARP and ASCL proteins [54] and, based on *in vitro* studies, there is no indication of DNA binding differences between ARP and ASCl proteins [55,56]. Therefore, these factors can regulate overlapping sets of genes as described for NEUROG3 and NEUROD1 [27] or for the proneural bHLH factors Xath5 and XNeuroD [57].

However, in other cases, the protein sequence can be crucial for the intrinsic properties of ARP/ASCL proteins acting as ‘cell fate determinants’ versus ‘cell differentiation factors.’ For example, in the context of muscle differentiation, the ‘cell differentiation gene’ *Myogenin (Myog)* is less efficient than the ‘cell fate determinant gene’ *Myogenic factor 5 (Myf5)* at remodeling chromatin and activating transcription at previously silent loci [58]. Such specificities can result from their interactions with specific cofactors. For example, functional divergence between Ato and Neurog proteins in *Drosophila* is encoded by three non-conserved residues in the basic domain of these bHLH which are responsible for the differential interaction with Senseless or MyT1 [59]. Such cofactors can affect the interaction of the bHLH with their DNA binding sites and/or modulate their transcriptional activity. Identifying such cofactors will be an important issue to understand better the transcriptional properties of ARP/ASCL factors.

The lack of pancreatic defects in the zebrafish *neurog3* mutant contrasts with the crucial function of NEUROG3 in the mouse. This raised the question whether the *neurog3* gene studied here is the actual ortholog of the murine *Neurog3* gene. This seems to be the case as extensive searches in the zebrafish genome revealed the presence of only two *neurogenin* genes in zebrafish, *neurog3* and *neurog1*, *neurog3* being the most closely related to murine and human *NEUROG3* and the locus displaying a conserved synteny with the region of human chromosome 10 containing *NEUROG3*[30]. As the zebrafish genome is almost completely sequenced [60], it is highly unlikely that another *neurogenin* gene exists in zebrafish but this possibility cannot be totally excluded. Rather, we show here that the crucial role of murine NEUROG3 in pancreatic endocrine cell differentiation is fulfilled in zebrafish by two bHLH transcription factors, Ascl1b and Neurod1. Indeed, the simultaneous inactivation of these two bHLH factors leads to a complete loss of all hormone-secreting cells, as in *Neurog3* null mice. The endocrine cells remain at the precursor stage as they still express the *ascl1b* transcripts but none of the later pancreatic transcription factors, such as *sox4b, pax6b, mnx1, isl1* and *arx*. During the second endocrine wave coming from the ventral bud, Ascl1b and Neurod1 probably also play an important role as we have observed a strong expression of these two factors in the intrapancreatic ducts after Notch inhibition.

This indicates that NEUROG3 is not the only cell fate determinant that can promote pancreatic endocrine cell differentiation. Even in the mouse, some rare endocrine cells can be produced in a NEUROG3-independent manner as Wang *et al*. reported a small, yet significant, number of glucagon expressing cells in the *Neurog3*-/- pancreas before e15.5 [61]. One could, therefore, consider differentiating stem cells into insulin-producing cells without the involvement of NEUROG3 but via another ARP/ASCL factor. Such an assumption is strengthened by the fact that in the murine stomach, it is ASCL1 which is required for the differentiation of all endocrine cell types [62] whereas NEUROG3 is only involved in the differentiation of a subset of these cells [63,64]. In contrast, ASCL1 is only expressed in a few cells of the murine pancreas and its knock-out does not disrupt the pancreatic endocrine differentiation [28]. These results highlight the diversity found in the selection of the ARP/ASCL factors involved in the determination and differentiation of the endocrine cells and show that the choice of these factors depends not only on the organ considered but also on the species.

## Methods

### Zebrafish maintenance, mutant lines and LY411575 treatment

Zebrafish (*Danio rerio*) were raised and cared for according to standard protocols [65]. Wild-type embryos from the AB strain were used and staged according to Kimmel (Kimmel *et al*., 1995). Homozygous mutants were obtained by mating heterozygous fish for the mind bomb (mib^ta52b^) allele [66] and for the neurog3^sa211^ allele (ZMP, Zebrafish Mutation Project [34]). The genotyping of neurog3^sa211^ embryos were done on DNA extracted from tails of WISH stained embryos by performing a PCR using primers 0230 (CCAACACATACCCAGTACCTC) and O233 (TGATTTGACCTCTGTCGAAC) followed by a nested PCR, 0231 (GCTTGCAAGAGGTAAGCATC) and O232 (TGTAATTATGCGCGAATCTC) and by subsequent sequencing of the PCR products. The LY411575 treatment was performed by incubating the embryos during the indicated period with a 10 μm LY411575 solution (Medchemexpress) and replacing the media every day.

### Search in the zebrafish genome of another neurog3 paralog gene

In order to discover all *neurog* genes present in the zebrafish genome, we screened the Zv9 Genome assembly using the ensembl genome browser [67] that covers 1.357 Gb in scaffolds placed on chromosomes 1 to 25 and 55 Mb in unassigned scaffolds. This zebrafish sequence is considered to be nearly complete. Indeed, out of a non-redundant set of 21,471 zebrafish cDNAs from ENA/Genbank, only 120 (0.6%) are not currently found in Zv9 (that is, these 120 do not have a match at 90% identity covering at least 10% of their length) [60]). Furthermore, we also screened the nucleotide collection (nt/nr) from the National Center for Biotechnology Information (NCBI) [68] that consists of GenBank + EMBL + DDBJ + PDB + RefSeq sequences. These databanks have been screened by the program ‘tblastn’ with the consensus amino acid sequence of the basic domain of vertebrate Neurog3 (RRXKANDRERNR) or of vertebrate Neurog (RRXKA(D/N)DRERNR), the amino acid sequence of the bHLH domain of murine NEUROG3 and of zebrafish Neurog3 and with the consensus amino acid sequence of the bHLH of all Neurog proteins

(RRXKANXRERXRMHXLNXALDXLRXXLPXFPXDXKLTKIETLRFAXNYIWALXXTXR). All these searches identified only 2 *neurog* genes in the zebrafish genome, *neurog3* (AF181996) and *neurog1* (AF017301).

### Riboprobes, whole-mount *in situ* hybridization

Antisense riboprobes were made by transcribing linearized cDNA clones with SP6, T7 or T3 polymerase using digoxigenin or DNP labeling mix (Roche) according to the manufacturer’s instructions. They were subsequently purified on NucAway spin columns (Ambion) and ethanol-precipitated. The zebrafish *ascl1a*[69], *ascl1b*[69], *sox4b*[37], *isl1*[70], *neurod1*[71], *pax6b*[72], *neurog3*[30], *atoh1a*[73], *atoh1b*[73], *neurod6a*[74], *neurod6b*[74], *atoh1c*[75], *atoh7*[76], *atoh8*[77], *mnx1*/*hb9*[42], *arx*[78], *pdx1*[79], *insulin*[79], *somatostatin 2 (PPS2)*[80], *ghrelin*[81] and *glucagon*[80] probes have been described elsewhere. The *neurod2*, *neurod4* and *neurog1* probes were obtained from Imagene clones MAGp998E0411982Q (Pst1, SP6), IRBOp991C31D (Pst1, T7) and IRBOp991BO232D (EcoRI, T7), respectively. Single whole-mount and double fluorescent *in situ* hybridizations were carried out as described [37].

For visible WISH, quantifications of the number of cells were performed by counting the cells under the microscope by focusing successively on each layer of stained cells. For that purpose, the NBT/BCIP staining was carefully monitored in order to avoid an overstaining which would have prevented us from visualizing the individual cell boundaries. This method has been validated by counting the number of *isl1*-expressing cells at 24 hpf after a visible WISH or after a fluorescent WISH analyzed by confocal microscopy; very similar results were found (a mean of 49 cells counted by the first method and of 47 by the second) (see Figure 7 in [50]). For fluorescent WISH, quantifications were performed by determining the volume occupied by specific cells in control and morphants, 100% being arbitrarily fixed as the mean of the volume occupied by these cells in control morphants. Cell volume has been calculated by the program Imaris (Bitplane) on the confocal images of each embryo.

### Whole-mount immunohistochemistry and TUNEL assay

WISH was performed using the following antibodies: rabbit polyclonal antibody against GFP (1:500; Chemicon), guinea pig polyclonal antibody against Insulin (1:500; Biomeda), rabbit polyclonal antibody against Somatostatin (1:1000; MP Biomedicals). The embryos were fixed with 2% formaldehyde in 0.1 M PIPES, 1.0 mM MgSO4 and 2 mM EGTA overnight at 4°C and washed three times with PBS/0.3% Triton X-100. The yolk was manually removed, the embryos permeabilized for one hour with PBS/1% Triton X-100 and then blocked for two hours in PBS/4% BSA/0.3% Triton X-100 at room temperature. Both primary and secondary antibodies were incubated overnight in the same blocking buffer. Washes were done with PBS/0.3% Triton X-100 for three hours to remove excess antibodies.

The fragmented DNA of apoptotic cells was identified by the TUNEL method, using the Apoptag Apoptosis *in situ* detection kit (Chemicon) in which the fragmented DNA fragments are labeled with digoxigenin-nucleotide, subsequently recognized by peroxidase coupled anti-digoxigenin antibody and revealed by incubating with tyramide-fluorescein isothiocyanate (FITC) substrate. Briefly, after the WISH, the endogenous peroxidases were inactivated by incubation for one hour in 3% H2O2 at room temperature, washed three times in PBS/1% Triton X-100 and incubated in Equilibration Buffer for one hour. The terminal deoxynucleotidyl transferase (TdT) reaction was performed by incubating the embryos in a mixture of 18 μl reaction buffer and 6 μl TdT enzyme overnight at 37°C. The reaction was stopped with 500 μl of stop/wash buffer for 10 minutes. Then, the embryos were rinsed 3 times for 10 minutes with PBS/0.3% Triton X-100. The detection was performed by incubating the embryos with 50 μl anti-digoxigenin conjugate (peroxidase) overnight at 4°C, rinsing three times for 10 minutes with PBS/0.3% Triton X-100 and incubating in 50 μl amplification reagent (Perkin Elmer). + tyramide-FITC (1/5000) for one hour at room temperature. After three 15-minute washes in PBS/0.3% Triton X-100, samples were mounted in Prolong (Invitrogen) and imaged.

### Imaging

Microscope pictures were obtained with an Olympus DP70 photocamera fixed on a BX60 Olympus microscope. Confocal imaging was performed using a Leica TCS SP2 inverted confocal laser microscope (Leica Microsystems, Germany) Digitized images were acquired using a 63X (NA 1.2) Plan-Apo water-immersion objective at 1024 X 1024 pixel resolution. For multicolor imaging, FITC was visualized by using an excitation wavelength of 488 nm and the emission light was dispersed and recorded at 500 to 535 nm. Cy3 was detected by using an excitation wavelength of 543 nm and the fluorescence emission was dispersed and recorded at 555 to 620 nm. The acquisition was set up to avoid any cross-talk of the two fluorescence emissions. Series of optical sections were carried out to analyze the spatial distribution of fluorescence, and for each embryo, they were recorded with a *Z*-step ranging between 1 and 2 μm. Image processing, including background subtraction, was performed with Leica software (version 2.5). Captured images were exported as TIFF and further processed using Adobe Photoshop and Illustrator CS2 for figure mounting.

### Morpholino design and injection

All the morpholinos (Mo) were designed by Gene Tools. The *neurog3* morpholinos are complementary to either the ATG (Mo1: 5′-GGATCTTGGAGTCATTCTCTTGCAA-3′) or to the 5′UTR (Mo2: 5′-GCTCGCTCAGTAAAACCGAGGTACT-3′). The *neurod1* morpholinos are complementary to either the ATG (Mo1: 5′-TTTCCTCGCTGTATGACTTCGTCAT-3′) or the 5′UTR (Mo2: 5′-CCTCTTACCTCAGTTACAATTTATA-3′). The *ascl1b* morpholino, as described by [44,45], is complementary to the ATG (5′-TCGTAGCGACGACAGTTGCCTCCAT-3′). A standard control Mo, having the sequence 5′-CCTCTTACCTCAGTTACAATTTATA 3′ has also been designed by Gene Tools in a way that it should have no target and no significant biological activity. A morpholino directed against *p53* mRNA was used to prevent nonspecific apoptosis [82], observed with the *ascl1b* morpholino. They were dissolved at a concentration of 3 μg/μl in 1× Danieau buffer containing 0.5% of rhodamine dextran (to follow the microinjection process) and microinjected at the 1 to 2 cells stage at a dose of 2.5 ng for Mo1-*neurog3*, 3 ng for Mo2-*neurog3*, 3 ng for Mo1 and Mo2 *neurod1* and 6 ng for Mo-*ascl1b* together with 3 ng Mo-*p53*. For the double knock-down of Ascl1b and Neurod1, a mix with 6 ng of Mo-*ascl1b*, 3 ng of Mo2-*neurod1* and 3 ng of Mo-*p53* was injected. Injected embryos were then grown in the presence of 0.003% 1-phenyl-2-thiourea until the desired stage, fixed overnight in 4% paraformaldehyde and stored in 100% methanol before analysis.

## Abbreviations

ARP: *Atonal* related protein; ASCL: *Achaete-Scute* like; bHLH: basic helix-loop-helix; BSA: Bovine serum albumin; dpf: days post fertilization; FITC: Fluorescein isothiocyanate; GFP: Green fluorescent protein; hpf: hours post fertilization; Mo: Morpholino; PBS: Phosphate-buffered saline; PCR: Polymerase chain reaction; s: somite; TUNEL: Terminal deoxynucleotidyl transferase dUTP nick end labeling; UTR: Untranslated region; WISH: Whole-mount *in situ* hybridization; wt: wild type.

## Competing interests

The authors declare that they have no competing interests.

## Authors’ contributions

LCF, BP and MLV designed the research; LCF, JLP, DGS, VVB and IM performed research and analyzed data; LCF, BP and MLV wrote the paper. All authors read and approved the final manuscript.

## Supplementary Material

Additional file 1: Figure S1 *neurog3* morphants do not display any apparent endocrine defects. WISH showing that the number of cells expressing *insulin* (**A-C**), *somatostatin* (**D-F**) and *glucagon* (**G-I**) is not changed in the Mo1-*neurog3* (B,E,H) or Mo2-*neurog3* (C,F,I) morphants compared to the Mo-control (A,D,G) morphants at 30 hpf. All views are ventral with the anterior part to the left. Click here for file

Additional file 2: Figure S2 *ascl1a, neurod6b, neurog1, atoh8* and *neurod6a* are expressed in the pancreatic area. WISH showing expression of *neurog1(***A***) ascl1a (***B***), neurod6b (***C***) neurod6a (***D***)* and *atoh8* (**E**) in the pancreatic region at the indicated stages. All views are ventral with anterior part to the left. Click here for file

Additional file 3: Figure S3 Mo1-*neurod1* and Mo2-*neurod1* morpholinos efficiently block the expression of GFP from the neurod1: egfp transgenic line. Lateral views of 26 to 30 hpf tg(neurod1:egfp) embryos injected with Mo1-*neurod1* (**A**), Mo2-*neurod1* (**B**) or control morpholinos analyzed by bright field illumination (A,B) or by epifluorescence illumination (A’,B’) showing that both morpholinos efficiently block the expression of GFP from the neurod1:egfp transgenic line [40] without disturbing the general morphology of the embryos. Click here for file

Additional file 4: Figure S4 TUNEL assays on control and morphant embryos. (**A–D**) Confocal image projections of 30 hpf and 40 hpf control and *neurod1* morphants after TUNEL labeling for apoptotic cells (in green) and immunodetection of *insulin* cells. The arrows highlight individual TUNEL^+^ cells in the neural tube. No TUNEL^+^ cells were found in the pancreatic region of control or *neurod1* morphants at analyzed stages. (**E–H**) Confocal image projections of 14S and 19S control and *ascl1b/neurod1* double morphants after TUNEL labeling for apoptotic cells (in red) and immunodetection of GFP cells. No TUNEL+ cells were found in the pancreatic region of control or morphants at analyzed stages. Somites 1 (S1) to somites 4 (S4) are shown on the panels **G** and **H**. Click here for file

Additional file 5: Figure S5 The double *ascl1b/neurod1* morphants do not display general developmental defects. Bright field views of *wt* embryos injected with Mo-*ascl1b* and Mo2-*neurod1* morpholinos (**A**) or control morpholinos (**B**) showing no general developmental defects in the double morphants at 22 hpf. Click here for file

## References

[B1] Desgraz R Bonal C Herrera PL β-cell regeneration: the pancreatic intrinsic faculty Trends Endocrinol Metab 2011 22 34 43 10.1016/j.tem.2010.09.004 21067943

[B2] Prince VE Kinkel MD Recent advances in pancreas development: from embryonic pathways to programming renewable sources of beta cells F1000 Biol Rep 2010 2 17 20445833 10.3410/B2-17 PMC2863342

[B3] Tuduri E Kieffer TJ Reprogramming gut and pancreas endocrine cells to treat diabetes Diabetes Obes Metab 2011 13 53 59 21824257 10.1111/j.1463-1326.2011.01446.x

[B4] Zaret KS Grompe M Generation and regeneration of cells of the liver and pancreas Science 2008 322 1490 1494 10.1126/science.1161431 19056973 PMC2641009

[B5] Stafford D Hornbruch A Mueller PR Prince VE A conserved role for retinoid signaling in vertebrate pancreas development Dev Genes Evol 2004 214 432 441 15322880 10.1007/s00427-004-0420-6

[B6] D’Amour KA Bang AG Eliazer S Kelly OG Agulnick AD Smart NG Moorman MA Kroon E Carpenter MK Baetge EE Production of pancreatic hormone-expressing endocrine cells from human embryonic stem cells Nat Biotechnol 2006 24 1392 1401 10.1038/nbt1259 17053790

[B7] Chung WS Andersson O Row R Kimelman D Stainier DY Suppression of Alk8-mediated Bmp signaling cell-autonomously induces pancreatic beta-cells in zebrafish Proc Natl Acad Sci U S A 2010 107 1142 1147 10.1073/pnas.0910205107 20080554 PMC2824271

[B8] Chung WS Shin CH Stainier DY Bmp2 signaling regulates the hepatic versus pancreatic fate decision Dev Cell 2008 15 738 748 10.1016/j.devcel.2008.08.019 19000838 PMC2610857

[B9] Chung WS Stainier DY Intra-endodermal interactions are required for pancreatic beta cell induction Dev Cell 2008 14 582 593 10.1016/j.devcel.2008.02.012 18410733 PMC2396532

[B10] Manfroid I Delporte F Baudhuin A Motte P Neumann CJ Voz ML Martial JA Peers B Reciprocal endoderm-mesoderm interactions mediated by fgf24 and fgf10 govern pancreas development Development 2007 134 4011 4021 10.1242/dev.007823 17942484

[B11] Naye F Voz ML Detry N Hammerschmidt M Peers B Manfroid I Essential roles of zebrafish bmp2a, fgf10, and fgf24 in the specification of the ventral pancreas Mol Biol Cell 2012 23 945 954 10.1091/mbc.E11-08-0664 22219376 PMC3290651

[B12] Stafford D Prince VE Retinoic acid signaling is required for a critical early step in zebrafish pancreatic development Curr Biol 2002 12 1215 1220 10.1016/S0960-9822(02)00929-6 12176331

[B13] Ober EA Field HA Stainier DY From endoderm formation to liver and pancreas development in zebrafish Mech Dev 2003 120 5 18 10.1016/S0925-4773(02)00327-1 12490292

[B14] Biemar F Argenton F Schmidtke R Epperlein S Peers B Driever W Pancreas development in zebrafish: early dispersed appearance of endocrine hormone expressing cells and their convergence to form the definitive islet Dev Biol 2001 230 189 203 10.1006/dbio.2000.0103 11161572

[B15] Field HA Dong PD Beis D Stainier DY Formation of the digestive system in zebrafish. II. Pancreas morphogenesis Dev Biol 2003 261 197 208 10.1016/S0012-1606(03)00308-7 12941629

[B16] Hesselson D Anderson RM Beinat M Stainier DY Distinct populations of quiescent and proliferative pancreatic beta-cells identified by HOTcre mediated labeling Proc Natl Acad Sci U S A 2009 106 14896 14901 10.1073/pnas.0906348106 19706417 PMC2736433

[B17] Wang Y Rovira M Yusuff S Parsons MJ Genetic inducible fate mapping in larval zebrafish reveals origins of adult insulin-producing beta-cells Development 2011 138 609 617 10.1242/dev.059097 21208992 PMC3026409

[B18] Esni F Ghosh B Biankin AV Lin JW Albert MA Yu X MacDonald RJ Civin CI Real FX Pack MA Ball DW Leach SD Notch inhibits Ptf1 function and acinar cell differentiation in developing mouse and zebrafish pancreas Development 2004 131 4213 4224 10.1242/dev.01280 15280211

[B19] Yee NS Lorent K Pack M Exocrine pancreas development in zebrafish Dev Biol 2005 284 84 101 10.1016/j.ydbio.2005.04.035 15963491

[B20] Zecchin E Filippi A Biemar F Tiso N Pauls S Ellertsdottir E Gnugge L Bortolussi M Driever W Argenton F Distinct delta and jagged genes control sequential segregation of pancreatic cell types from precursor pools in zebrafish Dev Biol 2007 301 192 204 10.1016/j.ydbio.2006.09.041 17059815

[B21] Kim W Shin YK Kim BJ Egan JM Notch signaling in pancreatic endocrine cell and diabetes Biochem Biophys Res Commun 2010 392 247 251 10.1016/j.bbrc.2009.12.115 20035712 PMC4152840

[B22] Bertrand N Castro DS Guillemot F Proneural genes and the specification of neural cell types Nat Rev Neurosci 2002 3 517 530 10.1038/nrn874 12094208

[B23] Rukstalis JM Habener JF Neurogenin3: a master regulator of pancreatic islet differentiation and regeneration Islets 2009 1 177 184 10.4161/isl.1.3.9877 21099270

[B24] Apelqvist A Li H Sommer L Beatus P Anderson DJ Honjo T Hrabe de Angelis M Lendahl U Edlund H Notch signalling controls pancreatic cell differentiation Nature 1999 400 877 881 10.1038/23716 10476967

[B25] Gradwohl G Dierich A LeMeur M Guillemot F neurogenin3 is required for the development of the four endocrine cell lineages of the pancreas Proc Natl Acad Sci U S A 2000 97 1607 1611 10.1073/pnas.97.4.1607 10677506 PMC26482

[B26] Grapin-Botton A Majithia AR Melton DA Key events of pancreas formation are triggered in gut endoderm by ectopic expression of pancreatic regulatory genes Genes Dev 2001 15 444 454 10.1101/gad.846001 11230152 PMC312631

[B27] Gasa R Mrejen C Lynn FC Skewes-Cox P Sanchez L Yang KY Lin CH Gomis R German MS Induction of pancreatic islet cell differentiation by the neurogenin-neuroD cascade Differentiation 2008 76 381 391 10.1111/j.1432-0436.2007.00228.x 17924961

[B28] Schwitzgebel VM Scheel DW Conners JR Kalamaras J Lee JE Anderson DJ Sussel L Johnson JD German MS Expression of neurogenin3 reveals an islet cell precursor population in the pancreas Development 2000 127 3533 3542 10903178 10.1242/dev.127.16.3533

[B29] Naya FJ Huang HP Qiu Y Mutoh H DeMayo FJ Leiter AB Tsai MJ Diabetes, defective pancreatic morphogenesis, and abnormal enteroendocrine differentiation in BETA2/neuroD-deficient mice Genes Dev 1997 11 2323 2334 10.1101/gad.11.18.2323 9308961 PMC316513

[B30] Wang X Chu LT He J Emelyanov A Korzh V Gong Z A novel zebrafish bHLH gene, neurogenin3, is expressed in the hypothalamus Gene 2001 275 47 55 10.1016/S0378-1119(01)00648-5 11574151

[B31] Fiuza UM Arias AM Cell and molecular biology of Notch J Endocrinol 2007 194 459 474 10.1677/JOE-07-0242 17761886

[B32] Fior R Henrique D “Notch-Off”: a perspective on the termination of Notch signalling Int J Dev Biol 2009 53 1379 1384 10.1387/ijdb.072309rf 19247952

[B33] Ehebauer M Hayward P Arias AM Notch, a universal arbiter of cell fate decisions Science 2006 314 1414 1415 10.1126/science.1134042 17138893

[B34] Zebrafish Mutant Resource http://www.sanger.ac.uk/Projects/D_rerio/zmp/

[B35] Wang Y Chen K Yao Q Zheng X Yang Z Phylogenetic analysis of zebrafish basic helix-loop-helix transcription factors J Mol Evol 2009 68 629 640 10.1007/s00239-009-9232-7 19449054

[B36] Dalgin G Ward AB Hao le T Beattie CE Nechiporuk A Prince VE Zebrafish mnx1 controls cell fate choice in the developing endocrine pancreas Development 2011 138 4597 4608 10.1242/dev.067736 21989909 PMC3190380

[B37] Mavropoulos A Devos N Biemar F Zecchin E Argenton F Edlund H Motte P Martial JA Peers B sox4b is a key player of pancreatic alpha cell differentiation in zebrafish Dev Biol 2005 285 211 223 10.1016/j.ydbio.2005.06.024 16055112

[B38] Soyer J Flasse L Raffelsberger W Beucher A Orvain C Peers B Ravassard P Vermot J Voz ML Mellitzer G Gradwohl G Rfx6 is an Ngn3-dependent winged helix transcription factor required for pancreatic islet cell development Development 2010 137 203 212 10.1242/dev.041673 20040487 PMC2799156

[B39] Itoh M Kim CH Palardy G Oda T Jiang YJ Maust D Yeo SY Lorick K Wright GJ Ariza-McNaughton L Weissman AM Lewis J Chandrasekharappa SC Chitnis AB Mind bomb is a ubiquitin ligase that is essential for efficient activation of Notch signaling by Delta Dev Cell 2003 4 67 82 10.1016/S1534-5807(02)00409-4 12530964

[B40] Obholzer N Wolfson S Trapani JG Mo W Nechiporuk A Busch-Nentwich E Seiler C Sidi S Sollner C Duncan RN Boehland A Nicolson T Vesicular glutamate transporter 3 is required for synaptic transmission in zebrafish hair cells J Neurosci 2008 28 2110 2118 10.1523/JNEUROSCI.5230-07.2008 18305245 PMC6671858

[B41] Collombat P Mansouri A Hecksher-Sorensen J Serup P Krull J Gradwohl G Gruss P Opposing actions of Arx and Pax4 in endocrine pancreas development Genes Dev 2003 17 2591 2603 10.1101/gad.269003 14561778 PMC218152

[B42] Wendik B Maier E Meyer D Zebrafish mnx genes in endocrine and exocrine pancreas formation Dev Biol 2004 268 372 383 10.1016/j.ydbio.2003.12.026 15063174

[B43] Delporte FM Pasque V Devos N Manfroid I Voz ML Motte P Biemar F Martial JA Peers B Expression of zebrafish pax6b in pancreas is regulated by two enhancers containing highly conserved cis-elements bound by PDX1, PBX and PREP factors BMC Dev Biol 2008 8 53 10.1186/1471-213X-8-53 18485195 PMC2409314

[B44] Nikolaou N Watanabe-Asaka T Gerety S Distel M Koster RW Wilkinson DG Lunatic fringe promotes the lateral inhibition of neurogenesis Development 2009 136 2523 2533 10.1242/dev.034736 19553285 PMC2709061

[B45] Amoyel M Cheng YC Jiang YJ Wilkinson DG Wnt1 regulates neurogenesis and mediates lateral inhibition of boundary cell specification in the zebrafish hindbrain Development 2005 132 775 785 10.1242/dev.01616 15659486

[B46] Pogoda HM von der Hardt S Herzog W Kramer C Schwarz H Hammerschmidt M The proneural gene ascl1a is required for endocrine differentiation and cell survival in the zebrafish adenohypophysis Development 2006 133 1079 1089 10.1242/dev.02296 16481349

[B47] Parsons MJ Pisharath H Yusuff S Moore JC Siekmann AF Lawson N Leach SD Notch-responsive cells initiate the secondary transition in larval zebrafish pancreas Mech Dev 2009 126 898 912 10.1016/j.mod.2009.07.002 19595765 PMC3640481

[B48] Ninov N Borius M Stainier DY Different levels of Notch signaling regulate quiescence, renewal and differentiation in pancreatic endocrine progenitors Development 2012 139 1557 1567 10.1242/dev.076000 22492351 PMC3317964

[B49] Manfroid I Ghaye A Naye F Detry N Palm S Pan L Ma TP Huang W Rovira M Martial JA Parsons MJ Moens CB Voz ML Peers B Zebrafish sox9b is crucial for hepatopancreatic duct development and pancreatic endocrine cell regeneration Dev Biol 2012 366 268 278 10.1016/j.ydbio.2012.04.002 22537488 PMC3364407

[B50] Binot AC Manfroid I Flasse L Winandy M Motte P Martial JA Peers B Voz ML Nkx6.1 and nkx6.2 regulate alpha- and beta-cell formation in zebrafish by acting on pancreatic endocrine progenitor cells Dev Biol 2010 340 397 407 10.1016/j.ydbio.2010.01.025 20122912

[B51] Wilson ME Scheel D German MS Gene expression cascades in pancreatic development Mech Dev 2003 120 65 80 10.1016/S0925-4773(02)00333-7 12490297

[B52] Jensen J Gene regulatory factors in pancreatic development Dev Dyn 2004 229 176 200 10.1002/dvdy.10460 14699589

[B53] Cau E Casarosa S Guillemot F Mash1 and Ngn1 control distinct steps of determination and differentiation in the olfactory sensory neuron lineage Development 2002 129 1871 1880 11934853 10.1242/dev.129.8.1871

[B54] Chien CT Hsiao CD Jan LY Jan YN Neuronal type information encoded in the basic-helix-loop-helix domain of proneural genes Proc Natl Acad Sci U S A 1996 93 13239 13244 10.1073/pnas.93.23.13239 8917575 PMC24077

[B55] Jarman AP Grau Y Jan LY Jan YN atonal is a proneural gene that directs chordotonal organ formation in the Drosophila peripheral nervous system Cell 1993 73 1307 1321 10.1016/0092-8674(93)90358-W 8324823

[B56] Powell LM Zur Lage PI Prentice DR Senthinathan B Jarman AP The proneural proteins Atonal and Scute regulate neural target genes through different E-box binding sites Mol Cell Biol 2004 24 9517 9526 10.1128/MCB.24.21.9517-9526.2004 15485919 PMC522279

[B57] Logan MA Steele MR Van Raay TJ Vetter ML Identification of shared transcriptional targets for the proneural bHLH factors Xath5 and XNeuroD Dev Biol 2005 285 570 583 10.1016/j.ydbio.2005.06.033 16112102

[B58] Gerber AN Klesert TR Bergstrom DA Tapscott SJ Two domains of MyoD mediate transcriptional activation of genes in repressive chromatin: a mechanism for lineage determination in myogenesis Genes Dev 1997 11 436 450 10.1101/gad.11.4.436 9042858

[B59] Quan XJ Denayer T Yan J Jafar-Nejad H Philippi A Lichtarge O Vleminckx K Hassan BA Evolution of neural precursor selection: functional divergence of proneural proteins Development 2004 131 1679 1689 10.1242/dev.01055 15084454

[B60] Howe K Clark MD Torroja CF Torrance J Berthelot C Muffato M Collins JE Humphray S McLaren K Matthews L McLaren S Sealy I Caccamo M Churcher C Scott C Barrett JC Koch R Rauch GJ White S Chow W Kilian B Quintais LT Guerra-Assunção JA Zhou Y Gu Y Yen J Vogel JH Eyre T Redmond S Banerjee R The zebrafish reference genome sequence and its relationship to the human genome Nature 2013 496 498 503 10.1038/nature12111 23594743 PMC3703927

[B61] Wang S Hecksher-Sorensen J Xu Y Zhao A Dor Y Rosenberg L Serup P Gu G Myt1 and Ngn3 form a feed-forward expression loop to promote endocrine islet cell differentiation Dev Biol 2008 317 531 540 10.1016/j.ydbio.2008.02.052 18394599 PMC2423199

[B62] Kokubu H Ohtsuka T Kageyama R Mash1 is required for neuroendocrine cell development in the glandular stomach Genes Cells 2008 13 41 51 18173746 10.1111/j.1365-2443.2007.01146.x

[B63] Jenny M Uhl C Roche C Duluc I Guillermin V Guillemot F Jensen J Kedinger M Gradwohl G Neurogenin3 is differentially required for endocrine cell fate specification in the intestinal and gastric epithelium EMBO J 2002 21 6338 6347 10.1093/emboj/cdf649 12456641 PMC136953

[B64] Lee CS Perreault N Brestelli JE Kaestner KH Neurogenin 3 is essential for the proper specification of gastric enteroendocrine cells and the maintenance of gastric epithelial cell identity Genes Dev 2002 16 1488 1497 10.1101/gad.985002 12080087 PMC186338

[B65] Westerfield M The Zebrafish Book: A Guide for the Laboratory Use of Zebrafish (Danio rerio) 1995 3 Eugene, OR: M. Westerfield

[B66] Haddon C Jiang YJ Smithers L Lewis J Delta-Notch signalling and the patterning of sensory cell differentiation in the zebrafish ear: evidence from the mind bomb mutant Development 1998 125 4637 4644 9806913 10.1242/dev.125.23.4637

[B67] Ensembl Genome Browser http://www.ensembl.org/Danio_rerio/

[B68] BLAST: Basic Local Alignment Search Tool http://blast.ncbi.nlm.nih.gov/Blast.cgi

[B69] Allende ML Weinberg ES The expression pattern of two zebrafish achaete-scute homolog (ash) genes is altered in the embryonic brain of the cyclops mutant Dev Biol 1994 166 509 530 10.1006/dbio.1994.1334 7813774

[B70] Korzh V Edlund T Thor S Zebrafish primary neurons initiate expression of the LIM homeodomain protein Isl-1 at the end of gastrulation Development 1993 118 417 425 8223269 10.1242/dev.118.2.417

[B71] Korzh V Sleptsova I Liao J He J Gong Z Expression of zebrafish bHLH genes ngn1 and nrd defines distinct stages of neural differentiation Dev Dyn 1998 213 92 104 10.1002/(SICI)1097-0177(199809)213:1<92::AID-AJA9>3.0.CO;2-T 9733104

[B72] Krauss S Johansen T Korzh V Moens U Ericson JU Fjose A Zebrafish pax[zf-a]: a paired box-containing gene expressed in the neural tube EMBO J 1991 10 3609 3619 1718739 10.1002/j.1460-2075.1991.tb04927.x PMC453092

[B73] Millimaki BB Sweet EM Dhason MS Riley BB Zebrafish atoh1 genes: classic proneural activity in the inner ear and regulation by Fgf and Notch Development 2007 134 295 305 10.1242/dev.02734 17166920

[B74] Liao J He J Yan T Korzh V Gong Z A class of neuroD-related basic helix-loop-helix transcription factors expressed in developing central nervous system in zebrafish DNA Cell Biol 1999 18 333 344 10.1089/104454999315394 10235116

[B75] Kani S Bae YK Shimizu T Tanabe K Satou C Parsons MJ Scott E Higashijima S Hibi M Proneural gene-linked neurogenesis in zebrafish cerebellum Dev Biol 2010 343 1 17 10.1016/j.ydbio.2010.03.024 20388506

[B76] Masai I Stemple DL Okamoto H Wilson SW Midline signals regulate retinal neurogenesis in zebrafish Neuron 2000 27 251 263 10.1016/S0896-6273(00)00034-9 10985346

[B77] Yao J Zhou J Liu Q Lu D Wang L Qiao X Jia W Atoh8, a bHLH transcription factor, is required for the development of retina and skeletal muscle in zebrafish PLoS One 2010 5 e10945 10.1371/journal.pone.0010945 20532172 PMC2880597

[B78] Miura H Yanazawa M Kato K Kitamura K Expression of a novel aristaless related homeobox gene ‘Arx’ in the vertebrate telencephalon, diencephalon and floor plate Mech Dev 1997 65 99 109 10.1016/S0925-4773(97)00062-2 9256348

[B79] Milewski WM Duguay SJ Chan SJ Steiner DF Conservation of PDX-1 structure, function, and expression in zebrafish Endocrinology 1998 139 1440 1449 10.1210/en.139.3.1440 9492081

[B80] Argenton F Zecchin E Bortolussi M Early appearance of pancreatic hormone-expressing cells in the zebrafish embryo Mech Dev 1999 87 217 221 10.1016/S0925-4773(99)00151-3 10495291

[B81] Pauls S Zecchin E Tiso N Bortolussi M Argenton F Function and regulation of zebrafish nkx2.2a during development of pancreatic islet and ducts Dev Biol 2007 304 875 890 10.1016/j.ydbio.2007.01.024 17335795

[B82] Robu ME Larson JD Nasevicius A Beiraghi S Brenner C Farber SA Ekker SC p53 activation by knockdown technologies PLoS Genet 2007 3 e78 10.1371/journal.pgen.0030078 17530925 PMC1877875

