# Supplementary figures and images for: Ascl1b and Neurod1, instead of Neurog3, control pancreatic endocrine cell fate in zebrafish
Source: BMC Biol. 2013 Jul 8;11:78. doi: 10.1186/1741-7007-11-78 (PMC3726459; doi:10.1186/1741-7007-11-78)

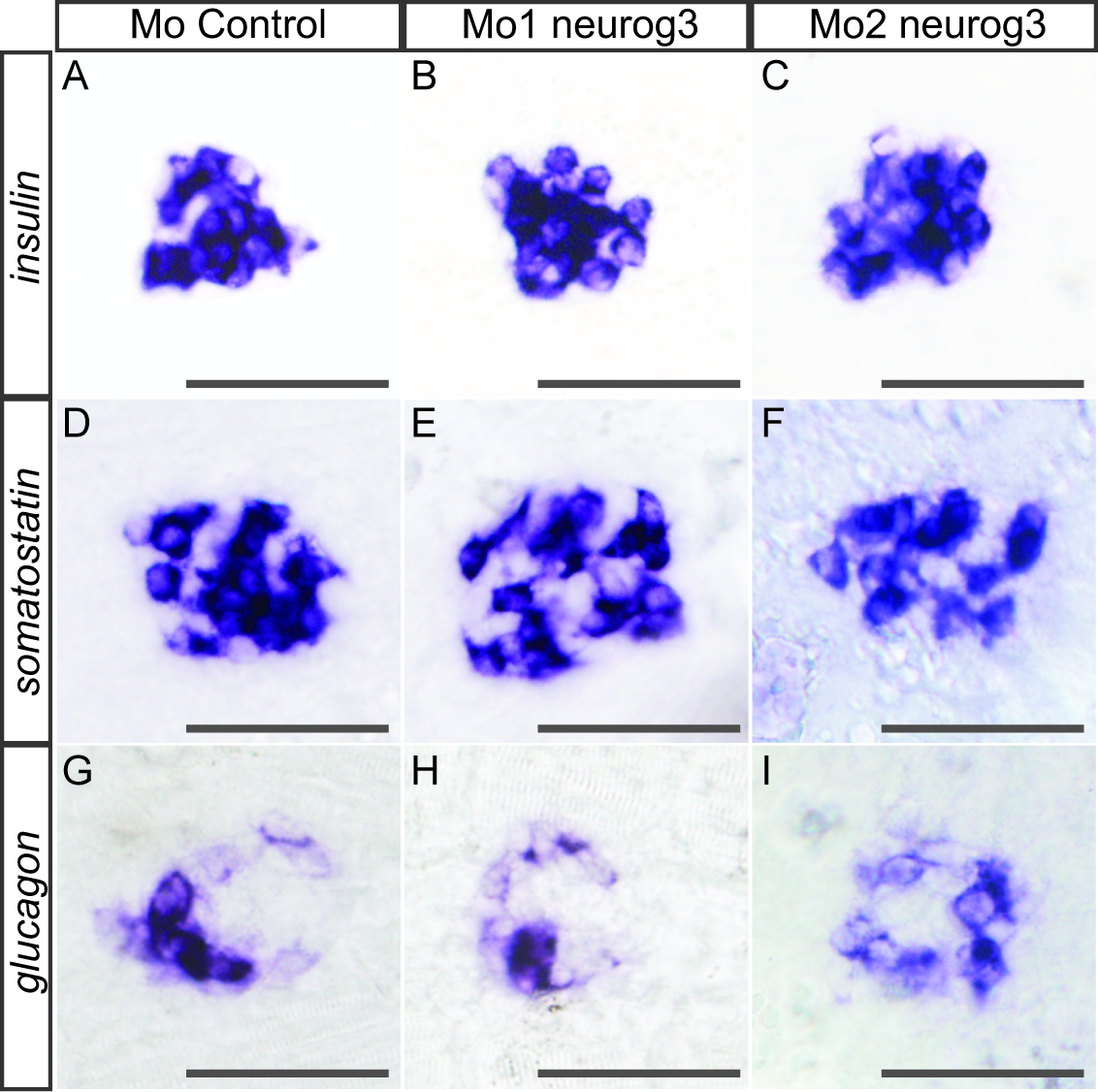

Supplement: Additional file 1: Figure S1 — neurog3 morphants do not display any apparent endocrine defects. WISH showing that the number of cells expressing insulin (A-C), somatostatin (D-F) and glucagon (G-I) is not changed in the Mo1-neurog3 (B,E,H) or Mo2-neurog3 (C,F,I) morphants compared to the Mo-control (A,D,G) morphants at 30 hpf. All views are ventral with the anterior part to the left. [file 1741-7007-11-78-S1.tiff]

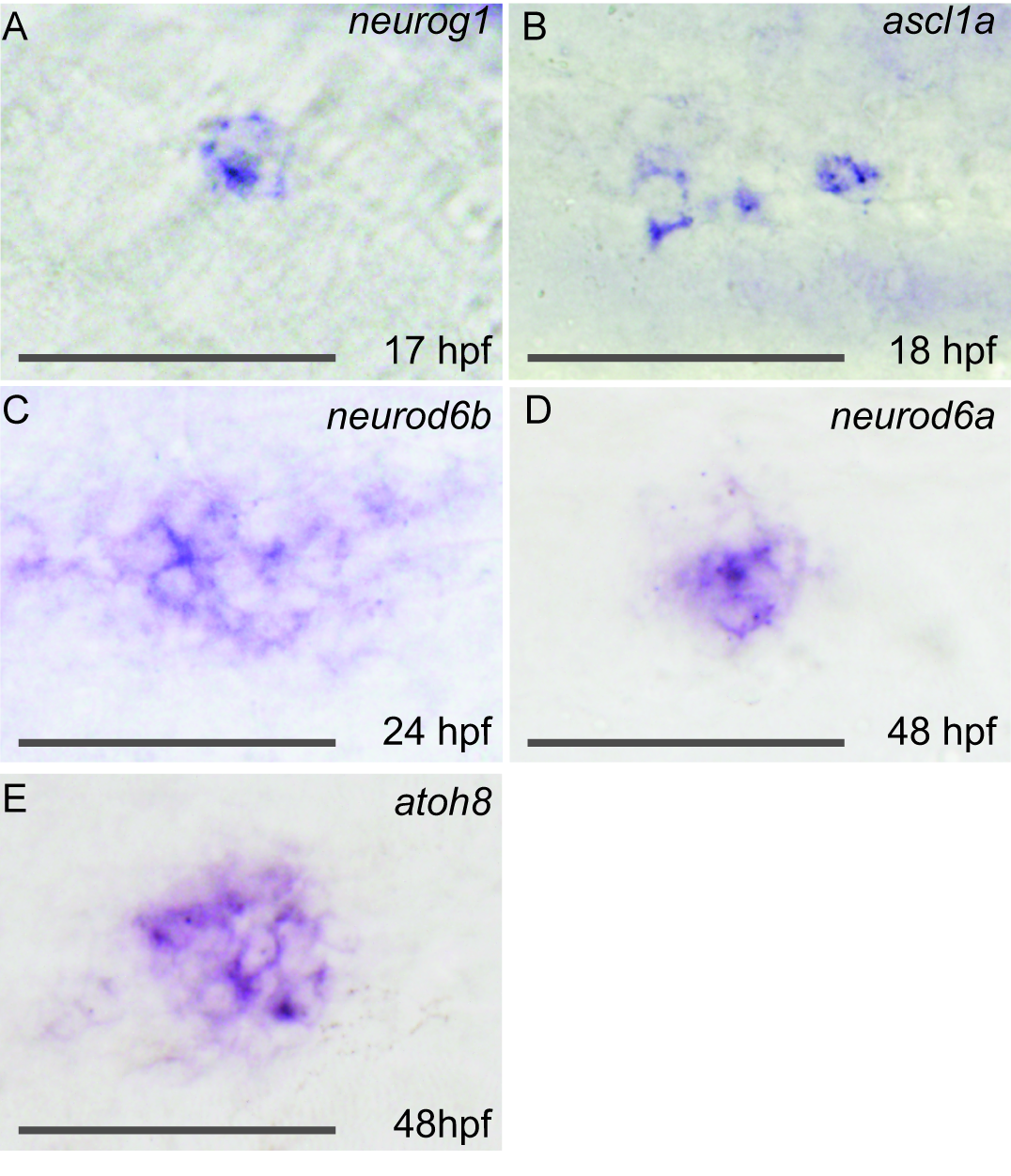

Supplement: Additional file 2: Figure S2 — ascl1a, neurod6b, neurog1, atoh8 and neurod6a are expressed in the pancreatic area. WISH showing expression of neurog1(A) ascl1a (B), neurod6b (C) neurod6a (D) and atoh8 (E) in the pancreatic region at the indicated stages. All views are ventral with anterior part to the left. [file 1741-7007-11-78-S2.tiff]

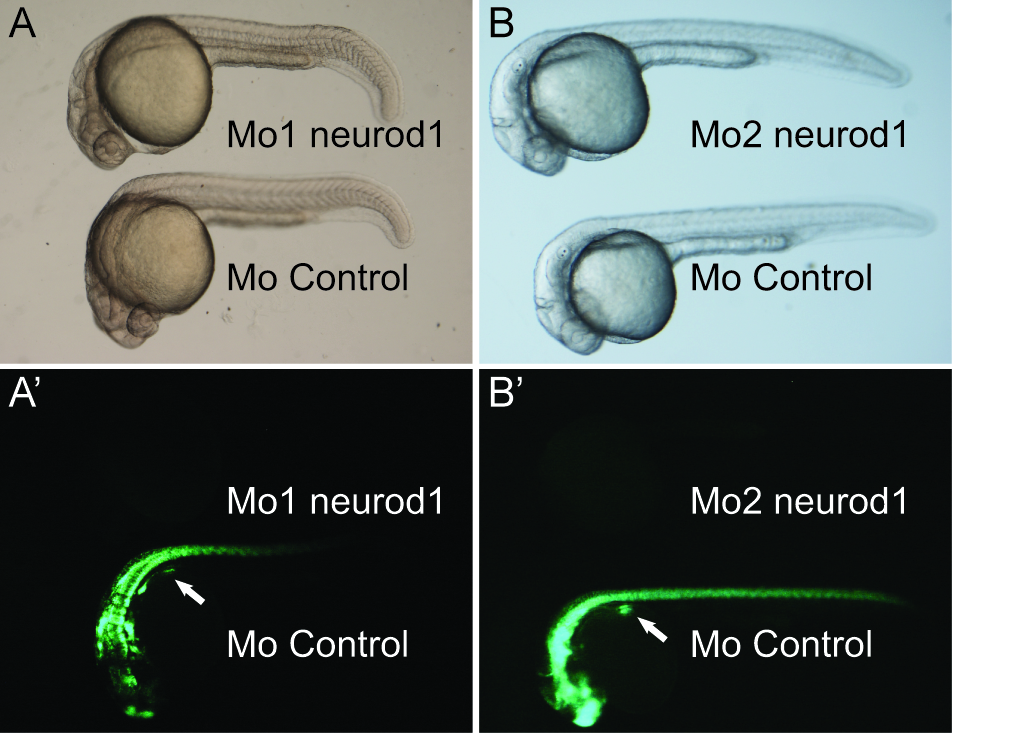

Supplement: Additional file 3: Figure S3 — Mo1-neurod1 and Mo2-neurod1 morpholinos efficiently block the expression of GFP from the neurod1: egfp transgenic line. Lateral views of 26 to 30 hpf tg(neurod1:egfp) embryos injected with Mo1-neurod1 (A), Mo2-neurod1 (B) or control morpholinos analyzed by bright field illumination (A,B) or by epifluorescence illumination (A’,B’) showing that both morpholinos efficiently block the expression of GFP from the neurod1:egfp transgenic line [40] without disturbing the general morphology of the embryos. [file 1741-7007-11-78-S3.tiff]

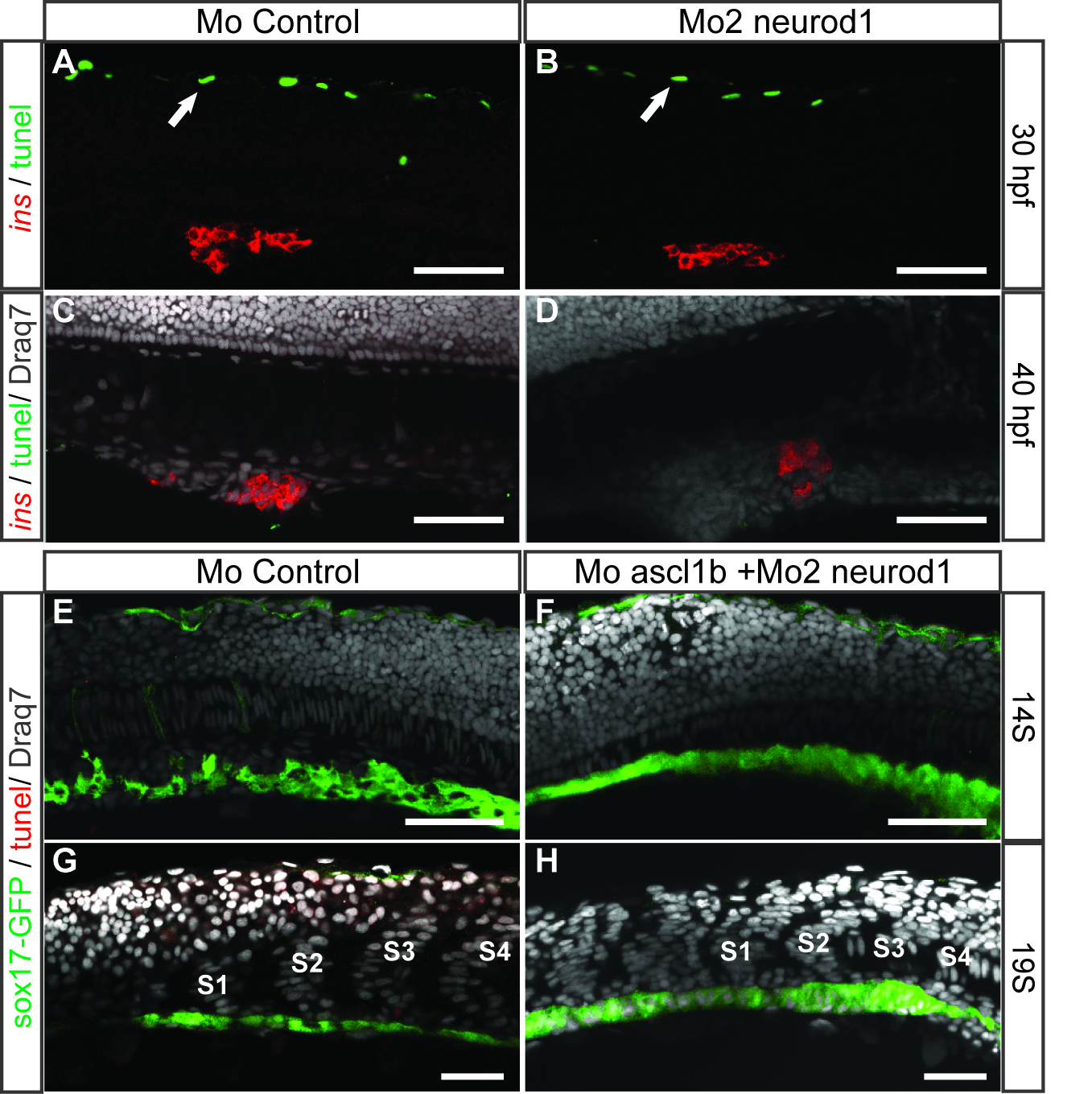

Supplement: Additional file 4: Figure S4 — TUNEL assays on control and morphant embryos. (A–D) Confocal image projections of 30 hpf and 40 hpf control and neurod1 morphants after TUNEL labeling for apoptotic cells (in green) and immunodetection of insulin cells. The arrows highlight individual TUNEL+ cells in the neural tube. No TUNEL+ cells were found in the pancreatic region of control or neurod1 morphants at analyzed stages. (E–H) Confocal image projections of 14S and 19S control and ascl1b/neurod1 double morphants after TUNEL labeling for apoptotic cells (in red) and immunodetection of GFP cells. No TUNEL+ cells were found in the pancreatic region of control or morphants at analyzed stages. Somites 1 (S1) to somites 4 (S4) are shown on the panels G and H. [file 1741-7007-11-78-S4.tiff]

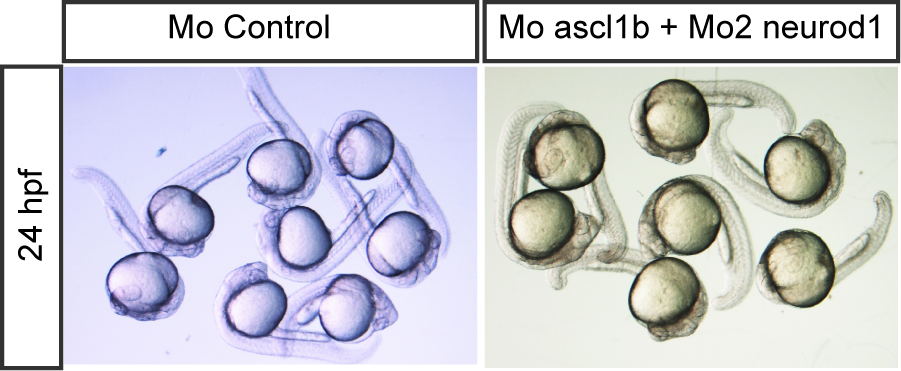

Supplement: Additional file 5: Figure S5 — The double ascl1b/neurod1 morphants do not display general developmental defects. Bright field views of wt embryos injected with Mo-ascl1b and Mo2-neurod1 morpholinos (A) or control morpholinos (B) showing no general developmental defects in the double morphants at 22 hpf. [file 1741-7007-11-78-S5.tiff]
